# A MARTX Toxin *rtxA* Gene Is Controlled by Host Environmental Signals through a CRP-Coordinated Regulatory Network in Vibrio vulnificus

**DOI:** 10.1128/mBio.00723-20

**Published:** 2020-07-28

**Authors:** Zee-Won Lee, Seung-Ho Hwang, Garam Choi, Kyung Ku Jang, Tae Hee Lee, Kyung Min Chung, Byoung Sik Kim, Sang Ho Choi

**Affiliations:** a National Research Laboratory of Molecular Microbiology and Toxicology, Department of Agricultural Biotechnology, Seoul National University, Seoul, Republic of Korea; b Center for Food Safety and Toxicology, Seoul National University, Seoul, Republic of Korea; c Department of Microbiology and Immunology, Jeonbuk National University Medical School, Jeonju, Jeonbuk, Republic of Korea; d Institute for Medical Science, Jeonbuk National University Medical School, Jeonju, Jeonbuk, Republic of Korea; e Department of Food Science and Engineering, Ewha Womans University, Seoul, Republic of Korea; University of Washington

**Keywords:** host environmental signals, MARTX toxin, regulatory network, *Vibrio vulnificus*

## Abstract

A multifunctional autoprocessing repeats-in-toxin (MARTX) toxin plays an essential role in the virulence of many pathogens, including a fulminating human pathogen Vibrio vulnificus. H-NS and HlyU repress and derepress expression of the MARTX toxin gene *rtxA* in V. vulnificus, respectively. However, little is known about other regulatory proteins and environmental signals involved in *rtxA* regulation. In this study, we found that a leucine-responsive regulatory protein (Lrp) activates *rtxA* by binding directly and specifically to the *rtxA* promoter, P*_rtxA_*. Phased hypersensitivity resulting from DNase I cleavage of the P*_rtxA_* regulatory region suggests that Lrp probably induces DNA bending in P*_rtxA_*. Lrp activates P*_rtxA_* independently of H-NS and HlyU, and leucine inhibits Lrp binding to P*_rtxA_* and reduces the Lrp-mediated activation. Furthermore, a cyclic AMP receptor protein (CRP) represses P*_rtxA_*, and exogenous glucose relieves the CRP-mediated repression. Biochemical and mutational analyses demonstrated that CRP binds directly and specifically to the upstream region of P*_rtxA_*, which presumably alters the DNA conformation in P*_rtxA_* and thus represses *rtxA*. Moreover, CRP represses expression of *lrp* and *hlyU* by binding directly to their upstream regions, forming coherent feed-forward loops with Lrp and HlyU. In conclusion, expression of *rtxA* is controlled by a regulatory network comprising CRP, Lrp, H-NS, and HlyU in response to changes in host environmental signals such as leucine and glucose. This collaborative regulation enables the elaborate expression of *rtxA*, thereby enhancing the fitness and pathogenesis of V. vulnificus during the course of infection.

## Abstract (precis)

A MARTX toxin, RtxA, is an essential virulence factor of many pathogens, including *Vibrio* species. H-NS and HlyU repress and derepress, respectively, *rtxA* expression of a life-threatening pathogen, Vibrio vulnificus. We found that Lrp directly activates *rtxA* independently of H-NS and HlyU, and leucine inhibits the Lrp-mediated activation of *rtxA*. Furthermore, we demonstrated that CRP represses *rtxA* but derepresses in the presence of exogenous glucose. CRP represses *rtxA* not only directly by binding to upstream of *rtxA* but also indirectly by repressing *lrp* and *hlyU*. This is the first report of a regulatory network comprising CRP, Lrp, H-NS, and HlyU, which coordinates the *rtxA* expression in response to environmental signals such as leucine and glucose during infection. This elaborate regulatory network will enhance the fitness of V. vulnificus and contribute to its successful infection within the host.

## INTRODUCTION

Multifunctional autoprocessing repeats-in-toxin (MARTX) toxins are large secreted virulence factors produced by various bacterial genera, including *Aeromonas*, *Xenorhabdus*, *Photorhabdus*, and *Vibrio* (1, 2). MARTX toxins are composed of the effector domains that have cytopathic effects on host cells and the N-terminal and C-terminal repeat-containing regions that form a pore in the host cell membrane for the translocation of the effector domains (3, 4). With potent cytotoxic activities toward host cells, these MARTX toxins have been highly associated with virulence of many pathogens, especially in *Vibrio* species such as Vibrio cholerae, Vibrio anguillarum, and Vibrio vulnificus (4–6). Notably, the ability of V. vulnificus to cause diseases is strongly linked to the production of the MARTX toxin RtxA encoded by the *rtxA* gene in the *rtxHCA* operon (7). RtxA triggers cytoskeletal rearrangement, bleb formation, and actin aggregation of host cells (8). Such changes result in apoptotic and necrotic cell death and enable V. vulnificus to invade the host bloodstream (8–10). Furthermore, RtxA contributes to the survival of the pathogen during infection by antagonizing the phagocytic activity of host immune cells (11, 12). Although the structural and functional features of the MARTX toxins have been elucidated in *Vibrio* species (3–5, 12–15), work on the regulatory mechanisms and environmental signals involved in their expression is still limited.

In many bacterial pathogens, the expression of virulence factors is tightly regulated by environmental signals that the pathogens may encounter during all stages of infection (16). Accordingly, pathogens have evolved numerous transcriptional regulatory proteins to sense these signals and regulate the expression of virulence genes within the host (17, 18). A leucine-responsive regulatory protein (Lrp), as a global regulator, controls the expression of virulence factors in pathogens such as Salmonella enterica serovar Typhimurium, Xenorhabdus nematophila, and V. cholerae (19–21). As a small nucleoid-structuring protein, Lrp binds to specific sequences in the large regions of a promoter and induces the bending or wrapping of DNA in a multimeric form to control the expression of target genes (22, 23). The transcriptional regulatory action of Lrp on target genes may be modulated by the binding of leucine, a small effector molecule known to affect the multimeric state of the protein (24, 25). Upon addition of leucine, the regulatory activity of Lrp can be enhanced, reversed, or unaffected, as represented in the different regulatory modes of Lrp for individual genes (24). As a result, Lrp regulates a variety of genes in response to changing conditions, such as the nutritional state of bacteria and host, and coordinates gene expression in cooperation with other regulatory proteins (19, 21, 24).

The cyclic AMP receptor protein (CRP) is a central regulator of carbon and energy metabolism, which makes the expression of virulence factors metabolically coordinated (26, 27). The availability of carbon and energy sources in the environment is sensed by the carbohydrate phosphotransferase system (PTS). In the absence of glucose, the enzyme IIA^glu^ of PTS remains phosphorylated and activates adenylate cyclase which synthesizes cyclic AMP (cAMP), resulting in an increase of intracellular cAMP levels (26). cAMP is a signaling molecule that has a fundamental role in global regulation of genes involved in multiple cellular processes, including catabolic metabolism (28). CRP forms a complex with cAMP (cAMP-CRP complex) and then binds DNA to control gene expression. Therefore, the genes regulated by the cAMP-CRP complex are expressed in response to nutrient availability (29). In this way, CRP coordinates the expression of genes related to metabolism and pathogenesis and thus ensures optimal growth and virulence factor production in bacteria under changing environments. Accordingly, expression of virulence factors, such as cholera toxin (CT) and toxin coregulated pilus (TCP) of V. cholerae, type 3 fimbriae of Klebsiella pneumoniae, and plasminogen activator protease of Yersinia pestis, is regulated by CRP and affected by exogenous glucose (30–32).

The opportunistic human pathogen V. vulnificus is a causative agent of foodborne diseases from mild gastroenteritis to primary septicemia (33, 34). Infection by V. vulnificus is characterized by rapid dissemination and severe tissue destruction, leading to high mortality rates (34, 35). Despite the significant role of RtxA in the pathogenesis of V. vulnificus (8–12), relatively little is known about regulation of *rtxA* expression. Only two regulatory proteins, a histone-like nucleoid-structuring protein (H-NS) and HlyU, have been reported to control expression of *rtxA*. H-NS represses expression of *rtxA* by directly binding to multiple AT-rich regions in the *rtxA* promoter, P*_rtxA_*. HlyU directly binds to P*_rtxA_* and induces *rtxA* expression by relieving binding of H-NS (36). In the present study, we first identified Lrp as a positive regulator of *rtxA* transcription that directly binds to specific sequences within P*_rtxA_*. Molecular genetic analysis revealed that Lrp-mediated activation of *rtxA* is independent of H-NS and HlyU. We investigated the effect of leucine on the regulatory mode of Lrp and found that leucine acts as an antagonist of the P*_rtxA_* activation by Lrp. Furthermore, CRP represses *rtxA* expression, and glucose alleviates repression of *rtxA* caused by CRP. Biochemical and mutational analyses demonstrated that CRP binds directly and specifically to upstream regions in the P*_rtxA_* regulatory region, which results in repression of *rtxA*. Interestingly, CRP also represses both *lrp* and *hlyU* by directly binding to their upstream regions, forming coherent feed-forward loops with Lrp and HlyU to regulate *rtxA*. Taken together, this study suggests that *rtxA* expression is elaborately regulated by host environmental signals, including leucine and glucose, through the CRP-coordinated regulatory network for the overall success of V. vulnificus during infection.

## RESULTS

### Lrp and CRP affect *rtxA* transcription in addition to H-NS and HlyU.

**FIG 1:**
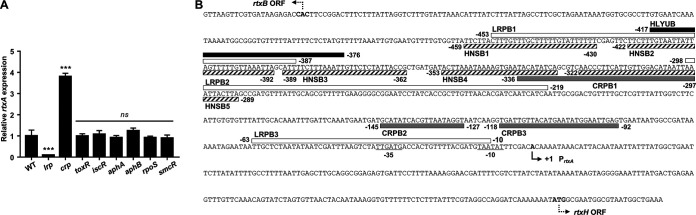
Expression of *rtxA* in V. vulnificus with different genetic background and sequence analysis of the P*_rtxA_* regulatory region. (A) Total RNAs were isolated from the wild type (WT) and isogenic mutants grown to an *A*_600_ of 0.5 and used to determine the *rtxA* transcript levels. The *rtxA* transcript levels were determined by qRT-PCR analyses, and the *rtxA* transcript level in the wild type was set at 1. Error bars represent the standard deviations (SD). Statistical significance was determined by the multiple comparisons after one-way analysis of variance (ANOVA) (***, *P* < 0.0005; *ns*, not significant relative to the wild type). *lrp*, *lrp* mutant; *crp*, *crp* mutant; *toxR*, *toxR* mutant; *iscR*, *iscR* mutant; *aphA*, *aphA* mutant; *aphB*, *aphB* mutant; *rpoS*, *rpoS* mutant; *smcR*, *smcR* mutant. (B) The DNA sequence between *rtxBDE* and *rtxHCA* is shown. The transcription start site of the *rtxHCA* operon is indicated in boldface type and by a solid bent arrow, and the positions of the putative −10 and −35 regions are underlined. The putative translational initiation codons of *rtxB* and *rtxH* are indicated in boldface type and by the dashed bent arrow, respectively. The sequences for the binding of Lrp (LRPB1, LRPB2, and LRPB3; white boxes) and CRP (CRPB1, CRPB2, and CRPB3; gray boxes) were determined in this study. The sequences for the binding of HlyU (HLYUB) and H-NS (HNSB1, HNSB2, HNSB3, HNSB4, and HNSB5) are indicated by black box and hatched boxes, respectively.

It has been reported that H-NS represses but HlyU derepresses *rtxA* transcription in V. vulnificus (36). In an effort to identify other transcription factor(s) associated with the *rtxA* regulation, the *rtxA* transcript levels were compared in the wild type and various isogenic mutants lacking transcription factors which are known to affect the expression of virulence genes in V. vulnificus (27, 37–43). The *rtxA* transcript level in the *lrp* mutant was significantly lower than that in the wild type (Fig. 1A), indicating that Lrp may act as a positive regulator of *rtxA* expression. In contrast, the *rtxA* transcript level in the *crp* mutant was substantially higher than that in the wild type (Fig. 1A), indicating that CRP may act as a negative regulator of *rtxA* expression. Expression of *rtxA* did not differ in the wild type and the mutants lacking ToxR, IscR, AphA, AphB, an alternative sigma factor RpoS (42), or a quorum-sensing master regulator SmcR (43) (Fig. 1A), suggesting that *rtxA* might not be regulated by those transcription factors under the conditions tested. This observation led us to further analyze the roles of Lrp and CRP in the transcription of *rtxA* at the molecular levels. Figure 1B shows the structure of the P*_rtxA_* regulatory region and the binding sites for the transcription factors involved in *rtxA* expression, as identified in this study and a previous report (36).

### Lrp activates *rtxA* expression by directly binding to P*_rtxA_*.

**FIG 2:**
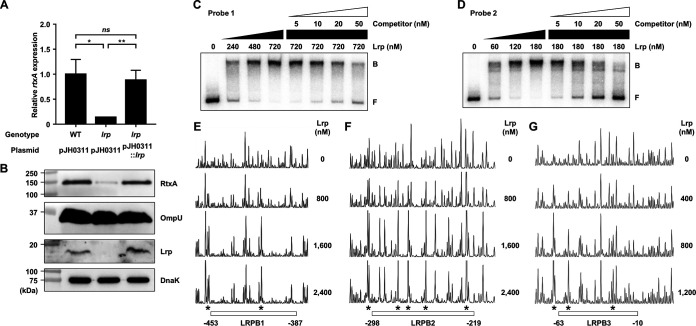
Effects of the *lrp* mutation on the *rtxA* expression and specific binding of Lrp to the P*_rtxA_* regulatory region. (A and B) Total RNAs and proteins were isolated from the V. vulnificus strains grown to an *A*_600_ of 0.5 and used to determine the *rtxA* transcript and RtxA, OmpU, Lrp, and DnaK protein levels. (A) The *rtxA* transcript levels were determined by qRT-PCR analyses, and the *rtxA* transcript level in the wild type was set at 1. Error bars represent the SD. Statistical significance was determined by the Student’s *t* test (**, *P* < 0.005; *, *P* < 0.05; *ns*, not significant). (B) The secreted levels of RtxA and OmpU (as an internal control) and cellular levels of Lrp and DnaK (as an internal control) were determined by Western blot analysis. Molecular size markers (Bio-Rad) are shown in kilodaltons. WT (pJH0311), wild type; *lrp* (pJH0311), *lrp* mutant; *lrp* (pJH0311::*lrp*), *lrp* complemented strain with pZW1818. (C and D) Each 452-bp DNA probe of the P*_rtxA_* regulatory region (probe 1 for panel C and probe 2 for panel D; 5 nM) was radioactively labeled and then incubated with increasing amounts of Lrp as indicated. For competition analysis, various amounts of the same but unlabeled DNA fragment were used as a self-competitor and added to the reaction mixture containing the 5 nM labeled DNA before the addition of 720 nM (C) or 180 nM (D) Lrp. The DNA-protein complexes were separated by electrophoresis. B, bound DNA; F, free DNA. (E to G) The same DNA probes of each P*_rtxA_* regulatory region (40 nM) were labeled with 6-FAM, incubated with increasing amounts of Lrp as indicated, and then digested with DNase I. The regions protected by Lrp are indicated by white boxes (LRPB1, LRPB2, and LRPB3), respectively. The nucleotides showing enhanced cleavage are indicated by asterisks. Nucleotide numbers shown are relative to the transcription start site of *rtxA*.

The *rtxA* transcript and RtxA protein levels were decreased in the *lrp* mutant and restored to the levels comparable to those in the wild type by introducing pZW1818 carrying a recombinant Lrp (Fig. 2A and B). These results confirmed that Lrp is a positive regulator of *rtxA* transcription. To examine whether Lrp directly binds to P*_rtxA_*, electrophoretic mobility shift assays (EMSAs) were performed. For this purpose, the 881-bp *rtxBDE-rtxHCA* intergenic region was divided into two 452-bp regions (referred to as probe 1 [positions −629 to −178] and probe 2 [positions −200 to +252] [positions from the transcription start site of *rtxHCA* {36}]; see Materials and Methods). The addition of Lrp to the radiolabeled DNA probe 1 and probe 2 resulted in a single retarded band of a DNA-Lrp complex in an Lrp concentration-dependent manner (Fig. 2C and D). The multiple bands observed with Lrp and probe 2 suggest either the binding of a multimeric Lrp to probe 2 or the multiple binding sites for Lrp in probe 2. Furthermore, Lrp at 720 nM was required for the full retardation of probe 1, while Lrp at 180 nM was sufficient for that of probe 2 (Fig. 2C and D). The results indicated that at least two binding sites for Lrp are present in the P*_rtxA_* regulatory region, where the upstream binding site(s) in probe 1 has relatively weaker affinity for Lrp binding than the downstream site(s) in probe 2. The same but unlabeled DNA fragment, which was used as a self-competitor, showed the competition for Lrp binding in a dose-dependent manner (Fig. 2C and D), confirming the specific binding of Lrp to P*_rtxA_*.

To identify the binding sequences for Lrp in the P*_rtxA_* regulatory region, DNase I protection assays were performed using the same DNA probes but labeled with 6-carboxyfluorescein (6-FAM). When Lrp was added to the DNA probes, Lrp largely protected three regions extending from positions −453 to −387 (Lrp-binding sequence 1 [LRPB1], centered at −420), −298 to −219 (LRPB2, centered at −258.5), and −63 to −10 (LRPB3, centered at −36.5), respectively, from DNase I digestion (Fig. 2E to G). Combined with the EMSA results, these results suggested that Lrp directly binds to LRPB1 and LRPB2 with similar binding affinities but binds to LRPB3 relatively strongly. Notably, within the regions protected by Lrp, a periodic pattern of reduced cleavage followed by short regions of enhanced cleavage was observed (Fig. 2E to G). This pattern is known as phased hypersensitivity and is consistent with the DNA bending by a multimeric Lrp (23), suggesting that Lrp multimers induce bending of the P*_rtxA_* regulatory region. Together, these results indicated that Lrp activates the *rtxA* transcription by binding directly and specifically to P*_rtxA_*.

### Lrp activates P*_rtxA_* independently of H-NS and HlyU.

**FIG 3:**
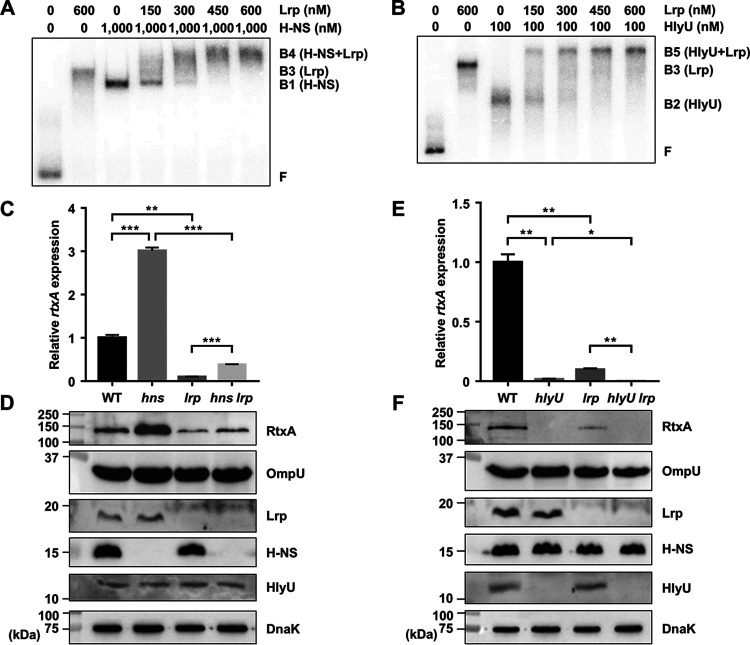
Lrp activates *rtxA* expression independently of H-NS and HlyU. (A and B) Interaction of Lrp with either H-NS (A) or HlyU (B) in the binding to P*_rtxA_*. A 452-bp DNA probe of the P*_rtxA_* regulatory region (probe 1; 5 nM) was radioactively labeled and then incubated with increasing amounts of Lrp in the presence of 1,000 nM H-NS (A) or 100 nM HlyU (B). The DNA-protein complexes were separated by electrophoresis. B1, DNA-H-NS complex; B2, DNA-HlyU complex; B3, DNA-Lrp complex; B4, DNA-H-NS-Lrp complex; B5, DNA-HlyU-Lrp complex; F, free DNA. (C to F) Total RNAs and proteins were isolated from the V. vulnificus strains grown to an *A*_600_ of 0.5 and used to determine the *rtxA* transcript and RtxA, OmpU, Lrp, H-NS, HlyU, and DnaK protein levels. (C and E) The *rtxA* transcript levels were determined by qRT-PCR analyses, and the *rtxA* transcript level in the wild type was set at 1. Error bars represent the SD. Statistical significance was determined by the Student’s *t* test (***, *P* < 0.0005; **, *P* < 0.005; *, *P* < 0.05). (D and F) The secreted levels of RtxA and OmpU (as an internal control) and cellular levels of Lrp, H-NS, HlyU, and DnaK (as an internal control) were determined by Western blot analysis. Molecular size markers (Bio-Rad) are shown in kilodaltons. WT, wild type; *hns*, *hns* mutant; *lrp*, *lrp* mutant; *hns lrp*, *hns lrp* double mutant; *hlyU*, *hlyU* mutant; *hlyU lrp*, *hlyU lrp* double mutant.

We observed that the binding sites of Lrp in the P*_rtxA_* regulatory region (LRPB1 and LRPB2) overlapped with those of H-NS and HlyU (36) (Fig. 1B). To understand how Lrp activates *rtxA* expression in the presence of H-NS or HlyU, we investigated whether Lrp interacts with H-NS or HlyU in the P*_rtxA_* regulatory region. To this end, EMSAs were performed using reaction mixtures containing the radiolabeled DNA probe 1 and a fixed concentration of either H-NS or HlyU with various amounts of Lrp. As the concentrations of Lrp increased, the band representing the DNA-H-NS complex (B1) was gradually retarded, generating the band representing the DNA-H-NS-Lrp complex (B4) (Fig. 3A). This result suggested that Lrp binds to the P*_rtxA_* regulatory region simultaneously with H-NS, rather than displaces H-NS. Similarly, EMSA with a fixed concentration of HlyU and increasing amounts of Lrp revealed that Lrp binds to the P*_rtxA_* regulatory region simultaneously with HlyU, rather than displaces HlyU (Fig. 3B).

We confirmed that H-NS negatively and HlyU positively regulate *rtxA* expression at the translational as well as transcriptional levels (Fig. 3C to F). The relationship of Lrp with either H-NS or HlyU in the regulation of *rtxA* was further investigated. As shown in Fig. 3C and D, the *rtxA* transcript and RtxA protein levels in the *hns lrp* double mutant decreased compared with those in the *hns* mutant. Furthermore, the extent of the decrease in the *rtxA* transcript and RtxA protein levels caused by the *lrp* mutation was similar in the wild type and *hns* mutant (Fig. 3C and D). These results indicated that Lrp activates *rtxA* expression in an independent manner with H-NS. Similarly, the *rtxA* transcript and RtxA protein levels in the *hlyU lrp* double mutant decreased compared with those in the *hlyU* mutant (Fig. 3E and F). The extent of the decrease in the *rtxA* transcript and RtxA protein levels carried by the *lrp* mutation was not affected by the presence of HlyU (Fig. 3E and F), indicating that Lrp activates *rtxA* expression in an independent manner with HlyU. Western blot analysis revealed that the cellular levels of Lrp, H-NS, and HlyU were not significantly affected by one another (Fig. 3D and F), suggesting that these transcriptional regulators function cooperatively to regulate *rtxA*, rather than sequentially in a regulatory cascade. Taken together, these results indicated that Lrp binds to the P*_rtxA_* regulatory region simultaneously with H-NS or HlyU and activates *rtxA* independently of H-NS and HlyU.

### Leucine inhibits Lrp binding to and activation of P*_rtxA_*.

**FIG 4:**
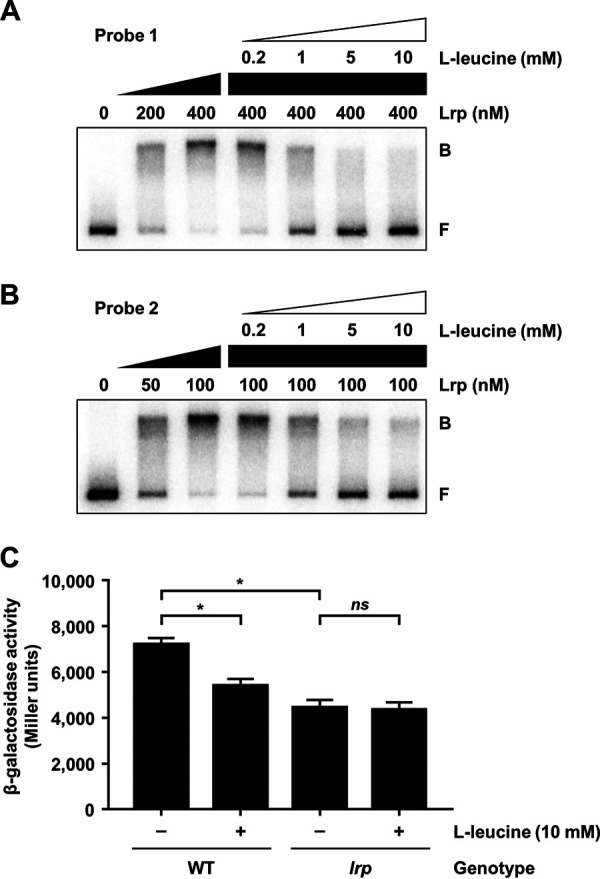
Effects of l-leucine on Lrp binding to and activation of P*_rtxA_*. (A and B) Each 452-bp DNA probe of the P*_rtxA_* regulatory region (probe 1 for panel A and probe 2 for panel B; 5 nM) was radioactively labeled and then incubated with increasing amounts of Lrp as indicated. Increasing amounts of l-leucine were added to the reaction mixture containing the 5 nM labeled DNA and 400 nM (A) or 100 nM (B) Lrp as indicated. The DNA-protein complexes were separated by electrophoresis. B, bound DNA; F, free DNA. (C) The V. vulnificus strains harboring the reporter plasmid with promoterless *lacZ* fused to P*_rtxA_* were grown to an *A*_600_ of 0.5 with or without 10 mM l-leucine. The β-galactosidase activities of the V. vulnificus cells were measured and expressed in Miller units. Error bars represent the SD. Statistical significance was determined by the Student’s *t* test (*, *P* < 0.05; *ns*, not significant). WT, *lacZ* mutant harboring the reporter plasmid; *lrp*, *lrp lacZ* double mutant harboring the reporter plasmid.

To investigate the effect of leucine on the regulatory mode of Lrp, we first examined whether leucine affects the DNA-binding activity of Lrp. EMSAs revealed that the addition of increasing amounts of leucine to the radiolabeled DNA probe 1 and probe 2 resulted in a concentration-dependent decrease of Lrp binding to DNA (Fig. 4A and B). The effects of different amino acids on the DNA-binding activity of Lrp were also examined. The addition of various amino acids decreased binding of Lrp in the order of leucine, methionine, isoleucine, and phenylalanine (see Fig. S1 in the supplemental material). In contrast, the addition of tryptophan and histidine did not alter the DNA-binding activity of Lrp (Fig. S1). Next, the P*_rtxA_* activity of V. vulnificus cells grown with or without leucine was determined *in vivo* using the *rtxA*-*lacZ* transcriptional fusion reporter. The presence of exogenous leucine significantly reduced the P*_rtxA_* activity in the wild-type strain but had no effect on the P*_rtxA_* activity in the *lrp* mutant (Fig. 4C). The results indicated that leucine exhibits an antagonistic effect on the P*_rtxA_* activity, and the effect is mediated by Lrp. Together, the results suggested that leucine inhibits Lrp binding to P*_rtxA_* and reduces P*_rtxA_* activation.

10.1128/mBio.00723-20.4 FIG S1 Effects of various amino acids on Lrp binding to the P*_rtxA_* regulatory region. (A and B) Each 452-bp DNA probe of the P*_rtxA_* regulatory region (probe 1 for panel A and probe 2 for panel B; 5 nM) was radioactively labeled and then incubated with 400 nM (A) or 100 nM (B) Lrp as indicated. l-Leucine (Leu), l-isoleucine (Ile), l-methionine (Met), l-histidine (His), l-phenylalanine (Phe), or l-tryptophan (Trp) at 5 mM were added to the reaction mixture containing the 5 nM labeled DNA and 400 nM (A) or 100 nM (B) Lrp. The DNA-protein complexes were separated by electrophoresis. B, bound DNA; F, free DNA. Download FIG S1, PDF file, 0.03 MB. Copyright © 2020 Lee et al. 2020 Lee et al. This content is distributed under the terms of the Creative Commons Attribution 4.0 International license.

### CRP represses but glucose derepresses P*_rtxA_*.

**FIG 5:**
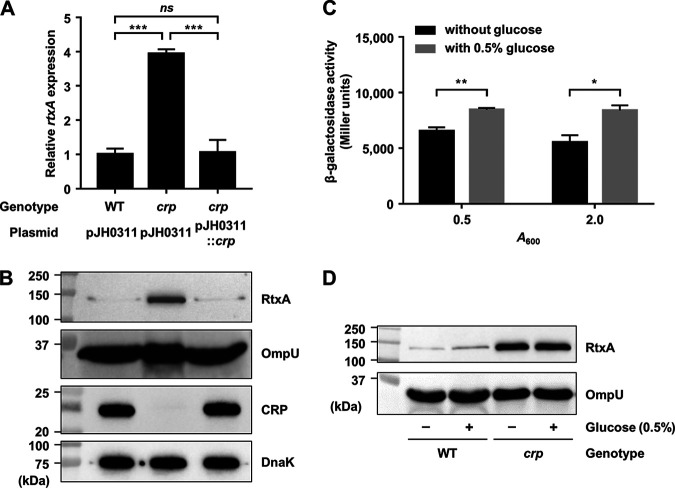
Effects of the *crp* mutation and glucose on *rtxA* expression. (A and B) Total RNAs and proteins were isolated from the V. vulnificus strains grown to an *A*_600_ of 0.5 and used to determine the *rtxA* transcript and RtxA, OmpU, CRP, and DnaK protein levels. (A) The *rtxA* transcript levels were determined by qRT-PCR analyses, and the *rtxA* transcript level in the wild type was set at 1. Error bars represent the SD. Statistical significance was determined by the Student’s *t* test (***, *P* < 0.0005; *ns*, not significant). (B) The secreted levels of RtxA and OmpU (as an internal control) and cellular levels of CRP and DnaK (as an internal control) were determined by Western blot analysis. WT (pJH0311), wild type; *crp* (pJH0311), *crp* mutant; *crp* (pJH0311::*crp*), *crp* complemented strain with pKK1502. (C) V. vulnificus *lacZ* mutant harboring the reporter plasmid with promoterless *lacZ* fused to P*_rtxA_* was grown to an *A*_600_ of 0.5 (for exponential phase) and 2.0 (for stationary phase) with or without 0.5% glucose. The β-galactosidase activity of the V. vulnificus cells was measured and expressed in Miller units. Error bars represent the SD. Statistical significance was determined by the Student’s *t* test (**, *P* < 0.005; *, *P* < 0.05). (D) The V. vulnificus strains were grown to an *A*_600_ of 0.5 with or without 0.5% glucose and used to determine RtxA and OmpU protein levels. The secreted levels of RtxA and OmpU were determined by Western blot analysis. WT, wild type; *crp*, *crp* mutant. Molecular size markers (Bio-Rad) are shown in kilodaltons in panels B and D.

Because the *crp* mutant showed significantly higher *rtxA* transcript level than the wild type (Fig. 1A), we investigated whether CRP acts as a negative regulator of *rtxA* expression. Consistent with the result, the *rtxA* transcript and RtxA protein levels were increased in the *crp* mutant and restored to levels comparable to those in the wild type by introducing pKK1502 carrying a recombinant CRP (Fig. 5A and B). These results indicated that expression of *rtxA* is negatively regulated by CRP. Furthermore, we also examined whether the expression of *rtxA* in V. vulnificus is increased by exogenous glucose using the *rtxA*-*lacZ* transcriptional fusion reporter. Indeed, in both exponential and stationary phases, P*_rtxA_* activity was higher in the cells grown with 0.5% glucose than that in the cells grown without glucose (Fig. 5C), indicating that the P*_rtxA_* activity is induced in the presence of exogenous glucose. We further investigated whether the production of RtxA is also increased upon the addition of exogenous glucose. As shown in Fig. 5D, the RtxA level was increased in wild-type V. vulnificus cells grown with 0.5% glucose compared with that in the cells grown without glucose. Moreover, the addition of exogenous glucose did not affect the production of RtxA in the *crp* mutant (Fig. 5D), confirming that the induction of *rtxA* in the presence of exogenous glucose is mediated by CRP. The combined results indicated that *rtxA* expression is repressed by CRP but derepressed by exogenous glucose.

### CRP directly binds to the upstream region of P*_rtxA_*.

**FIG 6:**
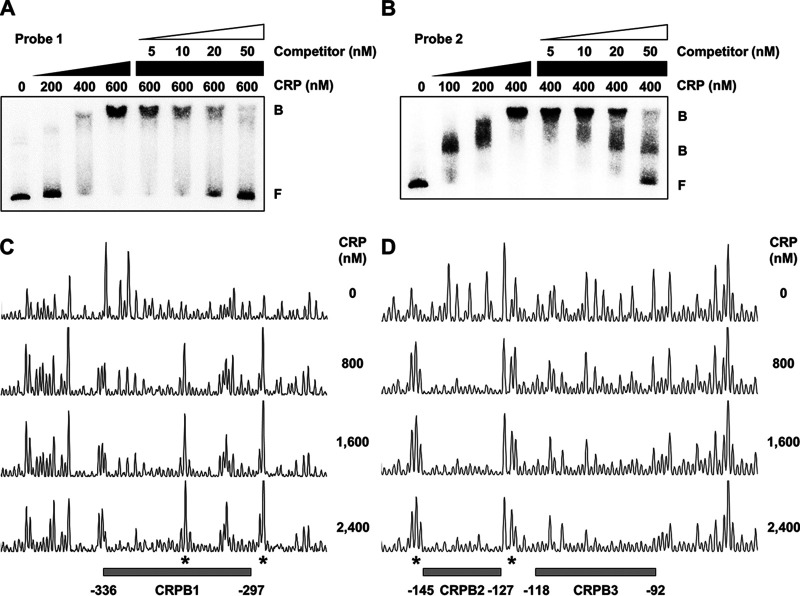
Specific binding of CRP to the P*_rtxA_* regulatory region. (A and B) Each 452-bp DNA probe of the P*_rtxA_* regulatory region (probe 1 for panel A and probe 2 for panel B; 5 nM) was radioactively labeled and then incubated with increasing amounts of CRP as indicated. For competition analysis, various amounts of the same but unlabeled DNA fragment were used as a self-competitor and added to the reaction mixture containing the 5 nM labeled DNA before the addition of 600 nM (A) or 400 nM (B) CRP. The DNA-protein complexes were separated by electrophoresis. B, bound DNA; F, free DNA. (C and D) The same DNA probes of each P*_rtxA_* regulatory region (40 nM) were labeled with 6-FAM, incubated with increasing amounts of CRP as indicated, and then digested with DNase I. The regions protected by CRP are indicated by gray boxes (CRPB1, CRPB2, and CRPB3), respectively. The nucleotides showing enhanced cleavage are indicated by asterisks. Nucleotide numbers shown are relative to the transcription start site of *rtxA*.

To examine whether CRP directly regulates *rtxA* by binding to P*_rtxA_*, EMSAs were performed. The addition of CRP to radiolabeled DNA probe 1 resulted in a single retarded band of a DNA-CRP complex in a CRP concentration-dependent manner (Fig. 6A). In contrast, the addition of CRP to radiolabeled DNA probe 2 resulted in two retarded bands (Fig. 6B), indicating that CRP binds to at least two binding sites in probe 2. The combined results suggested that at least three binding sites of CRP are present in the P*_rtxA_* regulatory region. The same but unlabeled DNA fragment, which was used as a self-competitor, showed the competition for CRP binding in a dose-dependent manner (Fig. 6A and B), confirming the specific binding of CRP to the P*_rtxA_* regulatory region. DNase I protection assays revealed that CRP protected two regions extending from positions −336 to −297 (CRP-binding sequence 1 [CRPB1], centered at −316.5) and −145 to −127 (CRPB2, centered at −136), respectively, from DNase I digestion (Fig. 6C and D). Upon an increase in the CRP concentrations, another additional region extending from positions −118 to −92 (CRPB3, centered at −105) was protected from DNase I digestion (Fig. 6D). Several nucleotides showed enhanced cleavage, which is frequently observed in the DNase I protection assay of CRP-binding sites (27, 44), indicating that CRP binding alters the DNA conformation in P*_rtxA_* (Fig. 6C and D). Combined with the EMSA results (Fig. 6A and B), these results suggested that CRP represses the expression of *rtxA* by directly binding to specific regions within P*_rtxA_*. It is noteworthy that all three binding sites of CRP are located in the upstream region of P*_rtxA_*, which is unusual for a negative regulator.

### Direct binding of CRP to P*_rtxA_* leads to the repression of *rtxA*.

**FIG 7:**
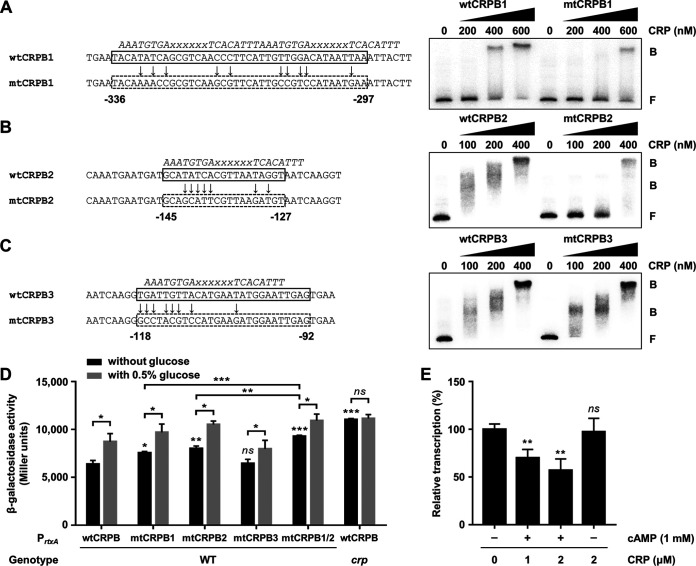
Effect of CRP binding on the P*_rtxA_* activity. (A to C) The CRP-binding sequences wild-type CRPB1 (wtCRPB1) (A), wtCRPB2 (B), and wtCRPB3 (C) in the P*_rtxA_* regulatory region are indicated by solid boxes. The mutated CRPBs (mtCRPBs) are indicated by dashed boxes, respectively, with the site-directed mutagenized nucleotides marked by arrows. The consensus sequences for CRP binding are indicated by italicized letters above the wtCRPBs. x, any nucleotide. Nucleotide numbers shown are relative to the transcription start site of *rtxA*. For EMSAs, each radiolabeled DNA probe of wtCRPB or mtCRPB (5 nM) was incubated with increasing amounts of CRP as indicated. The DNA-protein complexes were separated by electrophoresis. B, bound DNA; F, free DNA. (D) The V. vulnificus strains harboring reporter plasmids with promoterless *lacZ* fused to P*_rtxA_* carrying either wild-type or mutated CRPB as described in panels A to C were grown to an *A*_600_ of 0.5 with or without 0.5% glucose. The β-galactosidase activities of the V. vulnificus cells were measured and expressed in Miller units. Error bars represent the SD. Statistical significance was determined by the Student’s *t* test (***, *P* < 0.0005; **, *P* < 0.005; *, *P* < 0.05; *ns*, not significant relative to the wild-type strain with P*_rtxA_* carrying wtCRPB). WT, *lacZ* mutant harboring the reporter plasmid with promoterless *lacZ* fused to P*_rtxA_* carrying wtCRPB or mtCRPB; *crp*, *crp lacZ* double mutant harboring the reporter plasmid with promoterless *lacZ* fused to P*_rtxA_* carrying wtCRPB. (E) *In vitro* transcription of *rtxA*. An 881-bp *rtxBDE-rtxHCA* intergenic region spanning from positions −629 to +252 relative to the transcription start site of *rtxA* was used as a template DNA and transcribed *in vitro* with CRP in the presence or absence of cAMP as indicated. Relative transcription levels were determined using the heights of *rtxA* transcript peaks measured in arbitrary fluorescent units and expressed using the transcription level from the reaction without CRP as 100%. Error bars represent the SD from three independent experiments. Statistical significance was determined by Student’s *t* test (**, *P* < 0.005; *ns*, not significant relative to the transcription level from the reaction without CRP).

To investigate whether the CRP-binding sites located upstream of P*_rtxA_* are effective for CRP binding, we introduced mutations in the CRP-binding sequences as shown in Fig. 7A to C. First, the binding of CRP to P*_rtxA_* carrying the wild-type or each mutated CRP-binding sequence was examined. CRP bound to radiolabeled DNA probe 1 carrying the wild-type CRP-binding sequence 1, resulting in a single retarded band of the DNA-CRP complex in a CRP concentration-dependent manner (Fig. 7A, wtCRPB1). When the DNA probe carrying the mutated CRP-binding sequence 1 was used, however, the binding of CRP to DNA decreased, as a reduced amount of retarded bands was detected compared to the DNA probe with the wild-type CRP-binding sequence (Fig. 7A, mtCRPB1). Similar results were obtained with the DNA probe 2 carrying the wild-type or mutated CRP-binding sequence 2 (Fig. 7B, wtCRPB2 and mtCRPB2). The results indicated that the CRP-binding sites located upstream of P*_rtxA_* are effective for CRP binding. In contrast, the binding of CRP to the mutated CRP-binding sequence 3 was not significantly altered compared with that to the wild-type CRP-binding sequence (Fig. 7C, wtCRPB3 and mtCRPB3), suggesting that the binding of CRP to CRPB3 is less sequence specific than CRPB1 or CRPB2.

Next, we assessed the activity of P*_rtxA_* carrying the wild-type or each mutated CRP-binding sequence *in vivo* using the *rtxA*-*lacZ* transcriptional fusion reporters. Consistent with the EMSA results, the P*_rtxA_* activity in wild-type V. vulnificus cells was increased when either CRPB1 or CRPB2 was mutated but was not altered when CRPB3 was mutated (Fig. 7D). When both CRPB1 and CRPB2 were mutated (mtCRPB1/2), the P*_rtxA_* activity in wild-type V. vulnificus cells was higher than that in the cells with either mtCRPB1 or mtCRPB2, respectively (Fig. 7D). These results indicated that CRP binding to CRPB1 and CRPB2 is responsible for the *rtxA* repression *in vivo* and has an additive effect on the repression of *rtxA*. We obtained the same results using the strains that carry one copy of the wild-type or mutated CRP-binding sequence in P*_rtxA_* fused to promoterless *lacZ* on the chromosome (Fig. S2). Furthermore, the presence of exogenous glucose induced P*_rtxA_* activity in the wild-type strain, while it had no effect on P*_rtxA_* activity in the *crp* mutant (Fig. 7D). This result was consistent with the observation that induction of *rtxA* by exogenous glucose is mediated by CRP (Fig. 5D). Finally, the effect of CRP on the transcription of *rtxA* was analyzed by *in vitro* transcription assay. The transcription level of *rtxA* was decreased by CRP in the presence of cAMP but was not affected by CRP in the absence of cAMP (Fig. 7E and Fig. S3), confirming that CRP inhibits transcription of *rtxA* when cAMP is present. The combined results demonstrated that CRP represses P*_rtxA_* by directly binding to its upstream region.

10.1128/mBio.00723-20.5 FIG S2 Effect of CRP binding on the P*_rtxA_* activity using chromosomal *rtxA*-*lacZ* transcriptional fusion reporter strains. The V. vulnificus reporter strains with one copy of promoterless *lacZ* fused to P*_rtxA_* carrying either wild-type or mutated CRP-binding sequence (CRPB) as described in the legend to Fig. 7 were grown to an *A*_600_ of 0.5 with or without 0.5% glucose. The β-galactosidase activities of the V. vulnificus cells were measured and expressed in Miller units. Error bars represent the SD. Statistical significance was determined by the Student’s *t* test (***, *P* < 0.0005; **, *P* < 0.005; *, *P* < 0.05; *ns*, not significant relative to wild-type CRPB [wtCRPB]). Download FIG S2, PDF file, 0.02 MB. Copyright © 2020 Lee et al. 2020 Lee et al. This content is distributed under the terms of the Creative Commons Attribution 4.0 International license.

10.1128/mBio.00723-20.6 FIG S3 Effect of CRP on the *rtxA* transcription *in vitro*. An 881-bp *rtxBDE-rtxHCA* intergenic region spanning from positions −629 to +252 relative to the transcription start site of *rtxA* was used as a template DNA. The template DNA was incubated with CRP in the presence or absence of 1 mM cAMP prior to the addition of E. coli σ^70^-saturated RNA polymerase (Eσ^70^). The RNA transcript was reverse transcribed using a fluorescently (HEX) labeled primer and then mixed with a HEX-labeled DNA standard (Std). The resulting mixture was analyzed using an ABI 3730xl DNA analyzer (Applied Biosystems). The transcription start site of *rtxA* is indicated by asterisks. The signal from each electropherogram peak is measured in arbitrary fluorescence units along the *y* axis, and the transcript length is expressed in base pairs (bp) at the top of the panel. Download FIG S3, PDF file, 0.1 MB. Copyright © 2020 Lee et al. 2020 Lee et al. This content is distributed under the terms of the Creative Commons Attribution 4.0 International license.

### CRP directly represses *lrp* and *hlyU* expression.

**FIG 8:**
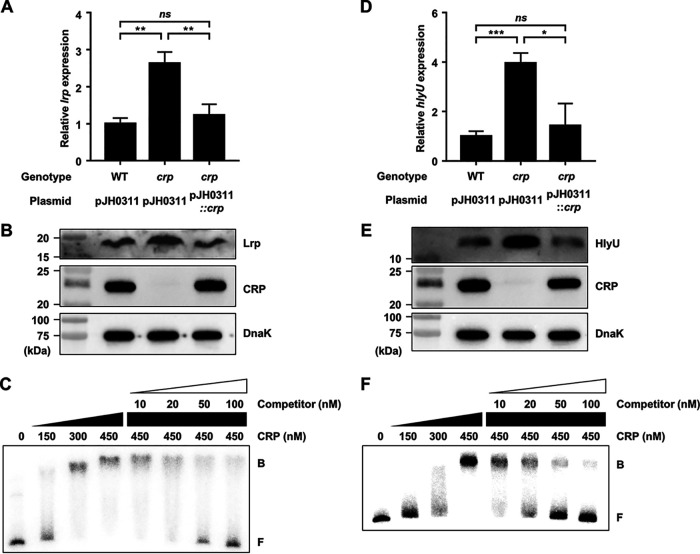
CRP represses the expression of *lrp* and *hlyU* by directly binding to their upstream regions. (A, B, D, and E) Total RNAs and proteins were isolated from the V. vulnificus strains grown to an *A*_600_ of 0.5 and used to determine the *lrp* and *hlyU* transcript and Lrp, HlyU, CRP, and DnaK protein levels. (A and D) The transcript levels of *lrp* (A) and *hlyU* (D) were determined by qRT-PCR analyses, and each transcript level in the wild type was set at 1. Error bars represent the SD. Statistical significance was determined by the Student’s *t* test (***, *P* < 0.0005; **, *P* < 0.005; *, *P* < 0.05; *ns*, not significant). (B and E) The cellular levels of Lrp, HlyU, CRP, and DnaK (as an internal control) were determined by Western blot analysis. Molecular size markers (Bio-Rad) are shown in kilodaltons. WT (pJH0311), wild type; *crp* (pJH0311), *crp* mutant; *crp* (pJH0311::*crp*), *crp* complemented strain with pKK1502. (C and F) A 424-bp DNA probe of the *lrp* upstream region (C) or a 572-bp DNA probe of the *hlyU* upstream region (F) was radioactively labeled and then incubated with increasing amounts of CRP as indicated. For competition analysis, various amounts of the same but unlabeled DNA fragment were used as a self-competitor and added to the reaction mixture containing the 5 nM labeled DNA before the addition of 450 nM CRP. The DNA-protein complexes were separated by electrophoresis. B, bound DNA; F, free DNA.

It is still possible that CRP indirectly regulates *rtxA* expression by modulating the cellular levels of other transcription factors. For example, CRP can negatively regulate *rtxA* expression by repressing the expression of Lrp or HlyU, the positive regulators of *rtxA*. Surprisingly, the *lrp* transcript and Lrp protein levels were significantly increased in the *crp* mutant and restored to the levels comparable to those in the wild type by complementation of the *crp* gene (Fig. 8A and B), indicating that CRP represses *lrp* expression. To examine whether CRP represses *lrp* by directly binding to its upstream region, EMSA was performed. The addition of CRP to the radiolabeled DNA resulted in a single retarded band of the DNA-CRP complex in a CRP concentration-dependent manner (Fig. 8C). The same but unlabeled DNA fragment, which was used as a self-competitor, showed the competition for CRP binding in a dose-dependent manner (Fig. 8C), confirming the specific binding of CRP to the upstream region of *lrp*. This result suggested that CRP directly represses *lrp* expression.

In a similar way, the *hlyU* transcript and HlyU protein levels were substantially increased in the *crp* mutant and restored to the levels comparable to those in the wild type by complementation of the *crp* gene (Fig. 8D and E), indicating that CRP also represses *hlyU* expression. Furthermore, EMSA revealed that CRP binds directly and specifically to the upstream region of *hlyU* (Fig. 8F). The combined results suggested that CRP directly represses expression of both *lrp* and *hlyU*, presumably resulting in the negative regulation of *rtxA*. Moreover, the cellular level of CRP was not altered by the mutation in *hns*, *lrp*, or *hlyU* (Fig. S4), suggesting that the expression of *crp* is not controlled by H-NS, Lrp, or HlyU. These results implied that CRP can also indirectly repress *rtxA* by forming coherent feed-forward loops with Lrp and HlyU. In conclusion, H-NS, HlyU, Lrp, and CRP constitute a regulatory network for *rtxA* transcription in V. vulnificus. CRP elaborately controls *rtxA* not only by directly binding to P*_rtxA_* but also by coordinating the components of the *rtxA* regulatory network through the repression of *lrp* and *hlyU*. This collaborative regulation perhaps contributes to the precise expression of *rtxA* in response to environmental signals such as leucine and glucose within the host.

10.1128/mBio.00723-20.7 FIG S4 Expression of *crp* is not affected by the *hns*, *lrp*, or *hlyU* mutation. (A and B) Total proteins were isolated from the V. vulnificus strains grown to an *A*_600_ of 0.5 as described in the legend to Fig. 3C to F and used to determine the CRP and DnaK protein levels. The cellular levels of CRP and DnaK (as an internal control) were determined by Western blot analysis. Molecular size markers (Bio-Rad) are shown in kilodaltons. WT, wild type; *hns*, *hns* mutant; *lrp*, *lrp* mutant; *hns lrp*, *hns lrp* double mutant; *hlyU*, *hlyU* mutant; *hlyU lrp*, *hlyU lrp* double mutant. Download FIG S4, PDF file, 0.02 MB. Copyright © 2020 Lee et al. 2020 Lee et al. This content is distributed under the terms of the Creative Commons Attribution 4.0 International license.

## DISCUSSION

In this study, Lrp was newly identified as a positive regulator of *rtxA* transcription in V. vulnificus (Fig. 1 and 2). Although the binding sites of Lrp in the P*_rtxA_* regulatory region overlapped with those of H-NS and HlyU (Fig. 1B), Lrp binds to P*_rtxA_* simultaneously with H-NS and HlyU (Fig. 3A and B). Consistent with this, Lrp activates *rtxA* expression independently of H-NS and HlyU (Fig. 3C to F). When acting as the sole transcriptional activator, Lrp can stimulate promoter activity by mediating protein-protein interactions and/or remodeling DNA structure (45). Based on our findings, one possible explanation for the Lrp-mediated activation of *rtxA* is that the formation of a multicomponent complex containing Lrp multimers and DNA bending could mediate the protein-protein interactions between Lrp and RNA polymerase (RNAP). Because the formation of the P*_rtxA_* DNA-Lrp complex is hindered in the presence of leucine (Fig. 4A and B), it is reasonable to hypothesize that leucine might inhibit the interactions between Lrp and RNAP and thus the activation of *rtxA*. Indeed, exogenous leucine significantly reduced P*_rtxA_* activity when Lrp was present (Fig. 4C). Accordingly, the regulatory mode of Lrp for *rtxA* in response to exogenous leucine is found to be the reciprocal mode, in which leucine inhibits the regulatory activity of Lrp (24).

Among a wide variety of environmental signals, nutrient availability is an important factor that pathogens monitor and integrate for the modulation of virulence gene expression (17, 18). In V. cholerae, the production of CT and TCP is negatively regulated by CRP, suggesting that intraintestinal glucose levels could affect the ability of the pathogen to colonize and cause diarrhea in the host (26). In enterotoxigenic Escherichia coli, the differential regulation of heat-labile toxin (LT) and heat-stable toxin (ST) by CRP also suggests that the glucose levels in the lumen of the small intestine might determine where and when each enterotoxin is expressed maximally (46). In this study, we found that the expression of *rtxA* in V. vulnificus is repressed by CRP (Fig. 5A and B). Because intracellular cAMP levels are tightly regulated by cAMP-synthesizing enzyme adenylate cyclase and cAMP-degrading enzyme cAMP phosphodiesterase in V. vulnificus (47), the addition of exogenous glucose would not completely deplete the intracellular cAMP levels. Thus, the presence of exogenous glucose might not fully alleviate the repression of *rtxA* as much as seen in the *crp* mutant. Despite this, the expression of *rtxA* is clearly induced by exogenous glucose (Fig. 5C and D), implying that the production of RtxA could increase in response to glucose in the host. It has been suggested that RtxA promotes the growth of V. vulnificus in the early stage of infection and dissemination of the bacteria from the small intestine to other organs in mice (10, 15). Therefore, it is tempting to speculate that, for successful pathogenesis, *rtxA* expression is favored in glucose-rich environments such as the duodenum and jejunum where carbohydrates are digested to monosaccharides, rather than in glucose-poor environments such as the ileum (46, 48). Induction of *rtxA* in the early stage of infection could provide the pathogen with the benefit of surviving from phagocytosis during infection (11, 12). Moreover, these findings are consistent with the observation that people whose glucose levels are higher due to diabetes or chronic liver disease are at high risk of infection by V. vulnificus (33).

EMSAs and DNase I protection assays revealed that CRP directly binds to specific sequences in the upstream region of P*_rtxA_* (Fig. 6), which is unusual as a negative regulator. Nevertheless, several transcription factors, including CRP, have been reported to bind upstream of promoters and repress transcription (29, 49). In V. cholerae, CRP negatively regulates the *rtxBDE* operon, encoding the components of the secretion system for RtxA, by directly binding to the upstream region of the promoter (29). Similarly, in enterotoxigenic E. coli, CRP directly represses the *eltA* gene encoding LT by binding to the upstream region of the promoter (46). V. vulnificus CRP also binds to the upstream region and represses P*_rtxA_*, and thus, we further examined whether the CRP-binding sites determined by the DNase I protection assays are effective for CRP binding (Fig. 7). Because H-NS binds to the sequences that overlapped with the CRP-binding sites in the upstream region of P*_rtxA_* (Fig. 1B), a deletion assay of the CRP-binding sites without deletion of the H-NS-binding sites was not possible. Therefore, site-directed mutational analyses of the CRP-binding sequences and *in vitro* transcription assay were performed to define whether CRP binding leads to the repression of P*_rtxA_*. The results demonstrate that CRP directly binds to the upstream region of P*_rtxA_* and represses *rtxA* expression (Fig. 7). One possible hypothesis for this unusual repression is that the upstream-bound CRP interacts with the C-terminal domains of the RNAP α subunit, thereby restraining RNAP from escaping from the initiation complex in P*_rtxA_* (49). This hypothesis can be supported by the enhanced cleavage that suggests the DNA conformation change in P*_rtxA_* by CRP binding, as shown in the DNase I protection assay results (Fig. 6C and D). In addition, CRP represses the expression of *lrp* and *hlyU* by directly binding to their upstream regions (Fig. 8). However, the mutation of *crp* in the absence of Lrp and HlyU still increased *rtxA* expression, and the extent of the increase in the *rtxA* transcript levels caused by the *crp* mutation was similar in the wild type and *hlyU lrp* double mutant (Fig. 5A; see also Fig. S5 in the supplemental material). This result suggests that the role of CRP in *rtxA* regulation would be mostly direct rather than indirect via controlling Lrp and HlyU. Again, this result supports the hypothesis that CRP binding might directly lead to the inhibition of promoter clearance of RNAP, instead of another hypothesis that CRP inhibits the *rtxA* activation mediated by Lrp and HlyU. Taken together, these results indicate that CRP plays a direct role in *rtxA* expression as a negative regulator and forms complex coherent feed-forward loops along with Lrp and HlyU for the coordinated regulation of *rtxA* (50).

10.1128/mBio.00723-20.8 FIG S5 Effect of *crp* mutation on the *rtxA* expression in the absence of Lrp and HlyU. Total RNAs were isolated from the *hlyU lrp* double and *hlyU lrp crp* triple mutants grown to an *A*_600_ of 0.5 and used to determine the *rtxA* transcript levels. The *rtxA* transcript levels were determined by qRT-PCR analyses, and the *rtxA* transcript level in the *hlyU lrp* double mutant was set at 1. Error bars represent the SD. Statistical significance was determined by the Student’s *t* test (**, *P* < 0.005). *hlyU lrp*, *hlyU lrp* double mutant; *hlyU lrp crp*, *hlyU lrp crp* triple mutant. Download FIG S5, PDF file, 0.01 MB. Copyright © 2020 Lee et al. 2020 Lee et al. This content is distributed under the terms of the Creative Commons Attribution 4.0 International license.

**FIG 9:**
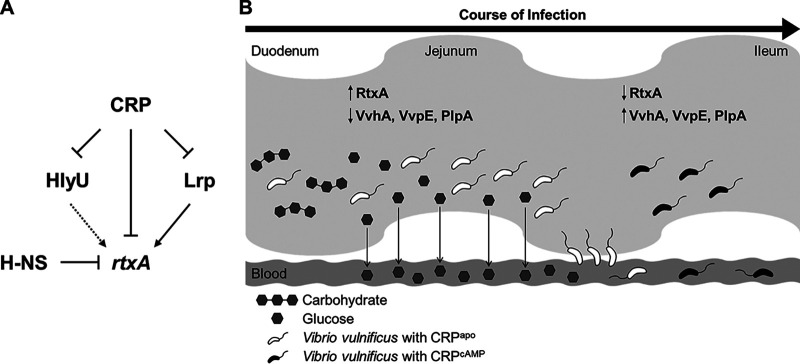
A regulatory network controlling the *rtxA* expression and a spatiotemporally differential expression of exotoxins in V. vulnificus. (A) A regulatory network comprising transcriptional regulators CRP, Lrp, HlyU, and H-NS controls *rtxA* expression. H-NS represses *rtxA*, and HlyU relieves the repression of H-NS. Lrp activates *rtxA*, while CRP represses *rtxA* directly and indirectly through the repression of *lrp* and *hlyU* in coherent feed-forward loops. (B) The V. vulnificus exotoxins may be expressed spatially and temporally as the bacteria move through the small intestine during the course of infection. In the early stage of infection, relatively high glucose levels in the duodenum and jejunum lead to the inactive form of CRP (CRP^apo^) and thereby prevent the CRP-mediated repression of *rtxA*. Thus, the expression of *rtxA* is predicted to be high, and the produced RtxA could contribute to the invasion of V. vulnificus from the small intestine to the bloodstream. In the later stage of infection, relatively low glucose levels in the ileum stimulate the synthesis of cAMP, resulting in the formation of active cAMP-CRP complex (CRP^cAMP^). Therefore, the expression of *rtxA* is decreased, and this decreased *rtxA* expression may be facilitated in response to other signals such as leucine at any rate (not shown). In contrast, the expression of *vvhA*, *vvpE*, and *plpA* is increased, causing further damage to host cells. This spatiotemporal and precise expression of the exotoxins will provide V. vulnificus with benefits in the pathogenesis during infection.

CRP, Lrp, H-NS, and HlyU constitute a complex regulatory network for *rtxA* transcription as depicted in Fig. 9A. H-NS represses *rtxA* by directly binding to multiple sites in the P*_rtxA_* regulatory region, while HlyU directly binds to P*_rtxA_* and relieves the repression of H-NS (36). Lrp directly activates *rtxA*, whereas CRP represses *rtxA* directly and indirectly through the repression of *lrp* and *hlyU* (Fig. 9A). Intriguingly, while CRP negatively regulates the expression of RtxA, CRP positively regulates the expression of other exotoxins, VvhA, VvpE, and PlpA, in V. vulnificus (27, 35, 42). As shown in Fig. 9B, this differential regulation is likely to determine which exotoxins are expressed spatially and temporally in V. vulnificus during the course of infection. For example, the expression of RtxA is predicted to be high in the early stage of infection such as in the upper small intestine and upon invasion into the bloodstream where glucose levels are relatively high. The production of RtxA could have an essential role in the invasion of V. vulnificus from the intestine to the bloodstream (8, 15), as well as in the survival of the pathogen from immune clearance (11, 51). The expression of *rtxA* will decrease in the later stage of infection where glucose levels are relatively low due to absorption by the enterocytes in the upper and mid small intestine (48) or consumption by the invading V. vulnificus in blood. The decreased expression of *rtxA* may be facilitated by responding to other signals such as leucine at any rate. Furthermore, the production of VvhA, VvpE, and PlpA in the later stage of infection might cause cell damage and accelerate the cell death process (8, 35, 52). Altogether, this spatiotemporal expression of virulence factors regulated by CRP will enhance the *in vivo* fitness of V. vulnificus. Coordinated regulation of *rtxA* by multiple transcription factors, including CRP and Lrp, enables the elaborate expression of *rtxA* in response to environmental and metabolic stimuli, further contributing to the successful infection of V. vulnificus within the host.

## MATERIALS AND METHODS

### Strains, plasmids, and culture conditions.

The strains and plasmids used in the present study are listed in Table S1 in the supplemental material. Unless stated otherwise, the V. vulnificus and E. coli strains were grown at 30°C in heart infusion (HI) medium supplemented with 2.5% (wt/vol) NaCl and at 37°C in Luria-Bertani (LB) medium, respectively. Growth of the V. vulnificus strains was monitored spectrophotometrically at 600 nm (*A*_600_). When necessary, antibiotics were added to the medium at the following concentrations: 3 μg/ml chloramphenicol and 2 μg/ml tetracycline for V. vulnificus and 20 μg/ml chloramphenicol and 10 μg/ml tetracycline for E. coli.

10.1128/mBio.00723-20.2 TABLE S1 Bacterial strains and plasmids used in this study. Download Table S1, DOCX file, 0.02 MB. Copyright © 2020 Lee et al. 2020 Lee et al. This content is distributed under the terms of the Creative Commons Attribution 4.0 International license.

### Generation and complementation of deletion mutants.

The isogenic *hns* mutant EJ151, *hlyU* mutant ZW141, *lacZ* mutant MO6Δ*lacZ*, and *crp* mutant DI0201 were constructed previously (35, 42, 53, 54) and used in this study (Table S1). For construction of an isogenic *lrp* mutant, the *lrp* gene was inactivated *in vitro* by deletion of the open reading frame (ORF) of *lrp* (324 of 495 bp) using the PCR-mediated linker-scanning method as described previously (44). Briefly, pairs of primers, LRPD-F1 and -R1 or LRPD-F2 and -R2, were used for amplification of the 5′ amplicon and 3′ amplicon, respectively (Table S2). The 324-bp deleted *lrp* gene was amplified by PCR using the mixture of both amplicons as the templates and LRPD-F1 and -R2 as the primers. The resulting 324-bp-deleted *lrp* gene was ligated into SpeI-SphI-digested pDM4 (55) to create pZW1817 (Table S1). Similarly, pGR1907 carrying the 501-bp-deleted *toxR* gene on pDM4 was constructed using the pairs of primers TOXRD-F1 and -R1 and TOXRD-F2 and -R2 (Table S2). E. coli S17-1 λ*pir* strain (56) containing pZW1817 was used as a conjugal donor to the V. vulnificus MO6-24/O wild type, *hns* mutant, *hlyU* mutant, or *lacZ* mutant to generate the *lrp* mutant ZW181, *hns lrp* double mutant ZW191, *hlyU lrp* double mutant ZW192, or *lrp lacZ* double mutant ZW193, respectively (Table S1). Similarly, E. coli S17-1 λ*pir* strain containing pGR1907 was used as a conjugal donor to the wild type to generate the *toxR* mutant GR192 (Table S1). E. coli S17-1 λ*pir* strain containing pBS0907 (57), which was constructed previously to carry a mutant allele of V. vulnificus *crp* on pDM4, was used as a conjugal donor to the *lacZ* mutant or *hlyU lrp* double mutant to generate the *crp lacZ* double mutant ZW194 or *hlyU lrp crp* triple mutant ZW195, respectively (Table S1). The conjugation and isolation of the transconjugants were conducted using the methods described previously (58).

10.1128/mBio.00723-20.3 TABLE S2 Oligonucleotides used in this study. Download Table S2, DOCX file, 0.04 MB. Copyright © 2020 Lee et al. 2020 Lee et al. This content is distributed under the terms of the Creative Commons Attribution 4.0 International license.

To complement the mutations, pKK1502 carrying the *crp* gene on the broad-host-range vector pJH0311 (59) was used in this study (Table S1) (35). Similarly, the *lrp* gene was amplified by PCR using a pair of primers LRPC-F and -R listed in Table S2 and cloned into pJH0311 to create pZW1818 (Table S1). The plasmids were transferred into the appropriate mutants by conjugation as described above.

### RNA purification and transcript analysis.

Total RNAs from the V. vulnificus strains were isolated using an RNeasy minikit (Qiagen, Valencia, CA). For quantitative reverse transcription-PCR (qRT-PCR), the concentrations of total RNAs were measured using a NanoDrop One^C^ spectrophotometer (Thermo Scientific, Waltham, MA), and cDNA was synthesized from 1 μg of the total RNAs using an iScript cDNA synthesis kit (Bio-Rad, Hercules, CA). Real-time PCR amplification of the cDNA was performed using a CFX96 real-time PCR detection system (Bio-Rad) with pairs of specific primers (Table S2), as described previously (60). Relative expression levels of the transcripts were calculated using the 16S rRNA expression level as an internal reference for normalization (60).

### Western blot analysis.

The V. vulnificus strains were harvested and fractionated into cells and supernatants by centrifugation. The supernatants were concentrated using Amicon Ultra-15 (cutoff, 30 kDa; Millipore, Burlington, MA). RtxA and OmpU in the supernatant concentrates were detected by Western blot analysis using mouse anti-V. vulnificus RtxA monoclonal antibody (61) and rabbit anti-V. vulnificus OmpU antibody (53). The cells were lysed using B-PER bacterial protein extraction reagent with enzymes (Thermo Fisher Scientific), and residual cell debris was removed by centrifugation to obtain clear cell lysates. Lrp, H-NS, HlyU, CRP, and DnaK in the clear cell lysates were detected by Western blot analysis using rabbit anti-V. vulnificus Lrp antibody (62), rabbit anti-V. vulnificus H-NS antibody (53), rabbit anti-V. vulnificus HlyU antibody (60), rabbit anti-V. vulnificus CRP antibody (35), and mouse anti-E. coli DnaK antibody (Enzo Life Science, Farmingdale, NY) as described previously (35, 60).

### Protein purification.

To overexpress H-NS, HlyU, and CRP, pKK1636 carrying the *hns* gene on pET-28a(+) (Novagen, Madison, WI) (53), pZW1610 carrying the *hlyU* gene on pProEX-HTa (Invitrogen, Carlsbad, CA) (60), and pHK0201 carrying the *crp* gene on pRSET A (Invitrogen) (42) were constructed previously and used in this study (Table S1). Similarly, the *lrp* gene was subcloned into pET-28a(+) using a pair of primers LRPP-F and -R listed in Table S2 to create pZW1903 (Table S1). The resulting His_6_-tagged Lrp, H-NS, HlyU, and CRP were expressed in E. coli BL21(DE3) and purified by affinity chromatography according to the manufacturer’s procedure (Qiagen).

### EMSA and DNase I protection assay.

For EMSA, the 881-bp *rtxBDE-rtxHCA* intergenic region containing P*_rtxA_* was divided into two 452-bp regions (positions −629 to −178 for probe 1 and positions −200 to +252 for probe 2 from the transcription start site of *rtxHCA* [36]). Each 452-bp DNA probe was amplified by PCR using unlabeled PrtxA_P1-F and [γ-^32^P]ATP-labeled PrtxA_P1-R or unlabeled PrtxA_P2-F and [γ-^32^P]ATP-labeled PrtxA_P2-R as primers, respectively (Table S2). Similarly, the 424-bp *lrp* upstream region and the 572-bp *hlyU* upstream region were amplified by PCR using unlabeled Plrp-F and [γ-^32^P]ATP-labeled Plrp-R or unlabeled PhlyU-F and [γ-^32^P]ATP-labeled PhlyU-R as primers, respectively (Table S2).

The radiolabeled DNA probes were incubated with the purified Lrp, H-NS, or HlyU for 0.5 h at 25°C in a 20-μl reaction mixture containing 1× Lrp binding buffer (50 mM Tris-Cl [pH 8.0], 20 mM KCl, 100 μg bovine serum albumin [BSA], 1 mM dithiothreitol [DTT], and 10% glycerol) and 0.1 μg of poly(dI-dC) (Sigma-Aldrich, St. Louis, MO) as described previously (21). Similarly, the DNA probes were incubated with the purified CRP for 0.5 h at 37°C in a 20-μl reaction mixture containing 1× CRP binding buffer (10 mM Tris-Cl [pH 7.9], 50 mM NaCl, 1 mM DTT, and 1 mM cAMP) and 0.1 μg of poly(dI-dC) as described previously (57). For competition analysis, the same but unlabeled DNA fragment was used as a self-competitor DNA. Electrophoretic analysis of the DNA-protein complexes was performed as described previously (60). When necessary, various concentrations of l-leucine were added to the reaction mixture before incubation.

For DNase I protection assay, the same DNA probes of each 452-bp P*_rtxA_* regulatory region were amplified by PCR using unlabeled PrtxA_P1-F and 6-FAM-labeled PrtxA_P1-R or unlabeled PrtxA_P2-F and 6-FAM-labeled PrtxA_P2-R as the primers, respectively (Table S2). The binding of purified Lrp or CRP to the labeled DNA was performed as described above. DNase I digestion of the DNA-protein complexes was performed as described previously (63). The digested DNA products were precipitated with ethanol, eluted in sterilized H_2_O, and then analyzed using an ABI 3730xl DNA analyzer (Applied Biosystems, Foster City, CA) with Peak Scanner software v1.0 (Applied Biosystems).

### Construction of an *rtxA-lacZ* transcriptional fusion reporter and β-galactosidase activity assay.

The 753-bp P*_rtxA_* regulatory region (positions −526 to +227 relative to the transcription start site of *rtxHCA*) was amplified using a pair of primers PrtxAZ-F and -R (Table S2) and then fused to promoterless *lacZ* (274 bp upstream from the translation start site of *lacZ*) of pRKΩlacZ (64) to create pZW1517 (Table S1). E. coli S17-1 λ*pir* strain containing pZW1517 was used as a conjugal donor to the *lacZ* mutant, *lrp lacZ* double mutant, or *crp lacZ* double mutant as described previously (58). The P*_rtxA_* activity of the V. vulnificus cells was determined by measuring the β-galactosidase activity. The β-galactosidase activity was determined by the chloroform/sodium dodecyl sulfate (SDS) method described previously by Miller (65).

### Site-directed mutagenesis of CRP-binding sequences.

The sequences of the wild-type CRP-binding sites (wtCRPB) determined by the DNase I protection assay were mutagenized using the PCR-mediated linker-scanning method with the mutagenic primers carrying the substituted nucleotides (Table S2) (66). For site-directed mutagenesis, pairs of primers, PrtxA_P1-F and CRPB1_mt-R or CRPB1_mt-F and PrtxA_P2-R, were used for amplification of the 5′ amplicon and 3′ amplicon of the mutated CRP-binding sequence 1 (mtCRPB1), respectively. Then, the mtCRPB1 was amplified by PCR using the mixture of both amplicons as the templates and PrtxA_P1-F and PrtxA_P2-R as the primers. Similarly, the mutated CRP-binding sequence 2 and 3 (mtCRPB2 and mtCRPB3) were created using the mutagenic primers listed in Table S2, respectively. All the mutations in the mtCRPBs were confirmed by DNA sequencing. The DNA probes of the P*_rtxA_* regulatory region carrying wtCRPB or mtCRPB were amplified using unlabeled and [γ-^32^P]ATP-labeled primers as described above and then used for EMSA.

For β-galactosidase activity assay, the reporter plasmid pZW1930 with promoterless *lacZ* fused to P*_rtxA_* carrying mtCRPB1 was constructed as described above (Table S1). Similarly, the reporter plasmids pZW1931, pZW1936, and pZW2001 with promoterless *lacZ* fused to P*_rtxA_* carrying either mtCRPB2, mtCRPB3, or mtCRPB1/2, respectively, were constructed (Table S1). E. coli S17-1 λ*pir* strain containing pZW1517, pZW1930, pZW1931, pZW1936, or pZW2001 was used as a conjugal donor to the *lacZ* mutant or *crp lacZ* mutant as described above. The activity of P*_rtxA_* carrying wtCRPB or mtCRPB in the V. vulnificus cells was determined by measuring the β-galactosidase activity as described above.

### *In vitro* transcription assay.

*In vitro* transcription was performed as described elsewhere (67). Briefly, the 881-bp *rtxBDE-rtxHCA* intergenic region containing P*_rtxA_* was amplified by PCR using PrtxA_P1-F and PrtxA_P2-R as primers (Table S2). One microgram of the template DNA was incubated with the purified CRP in the presence or absence of 1 mM cAMP for 0.5 h at 37°C in a 36-μl reaction mixture containing RNA polymerase reaction buffer (New England BioLabs [NEB]). Then, the mixture was supplemented with nucleotide triphosphate (NTP) mixture (0.5 mM each NTP), 40 units of RNase inhibitor (Invitrogen), and 1 unit of E. coli σ^70^-saturated RNA polymerase (Eσ^70^) (NEB), and incubated for 1 h at 37°C. The template DNA was digested with 1 unit of DNase I (Promega, Madison, WI) for 0.5 h at 37°C, and the RNA transcript was purified using an RNeasy MinElute Cleanup kit (Qiagen). The purified RNA was annealed with the 6-carboxy-2,4,4,5,7,7-hexachlorofluorescein (HEX)-labeled PrtxA_ivt-R (Table S2) and then extended using SuperScript IV reverse transcriptase (Invitrogen) for 1 h at 42°C according to the manufacturer’s procedure. A 389-bp HEX-labeled DNA standard generated with unlabeled PrtxA_P2-F and HEX-labeled PrtxA_ivt-R (Table S2) was added to each sample to a final concentration of 0.1 ng/μl. The samples were analyzed using an ABI 3730xl DNA analyzer (Applied Biosystems) with Peak Scanner software v1.0 (Applied Biosystems) as described above.

### Data analyses.

Averages and standard deviations (SD) were calculated from at least three independent experiments. Statistical analyses were performed by the Student’s *t* test or one-way analysis of variance (ANOVA) as indicated in the figure legends using GraphPad Prism 7.0 (GraphPad Software, San Diego, CA).

10.1128/mBio.00723-20.1 TEXT S1 Supplemental methods. Construction of chromosomal *rtxA*-*lacZ* transcriptional fusion reporter strains. Download Text S1, DOCX file, 0.02 MB. Copyright © 2020 Lee et al. 2020 Lee et al. This content is distributed under the terms of the Creative Commons Attribution 4.0 International license.
