# Supplementary figures and images for: A MARTX Toxin rtxA Gene Is Controlled by Host Environmental Signals through a CRP-Coordinated Regulatory Network in Vibrio vulnificus
Source: mBio. 2020 Jul 28;11(4):e00723-20. doi: 10.1128/mBio.00723-20 (PMC7387792; doi:10.1128/mBio.00723-20)

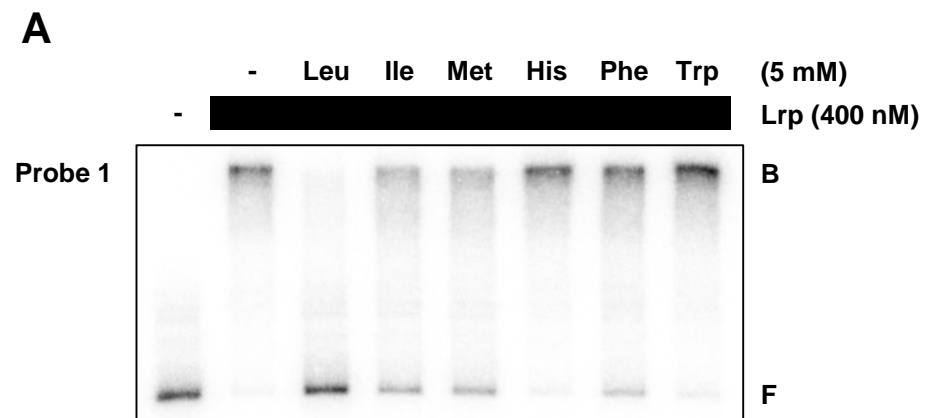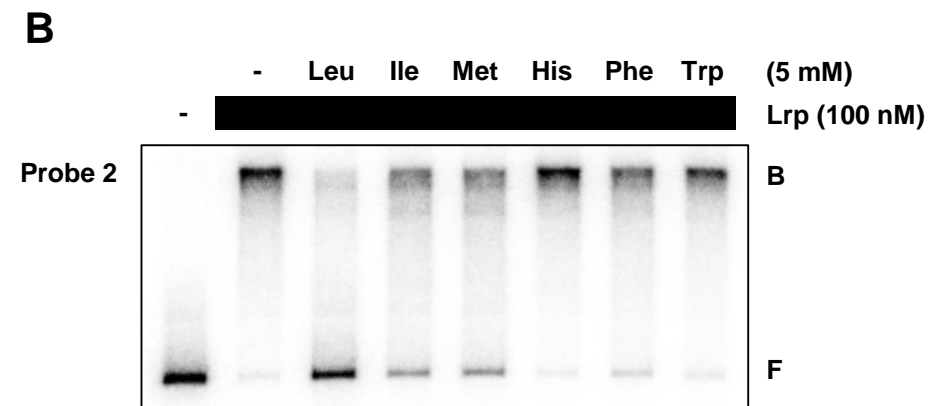

Supplement: FIG S1 [file mBio.00723-20-sf001.pdf]

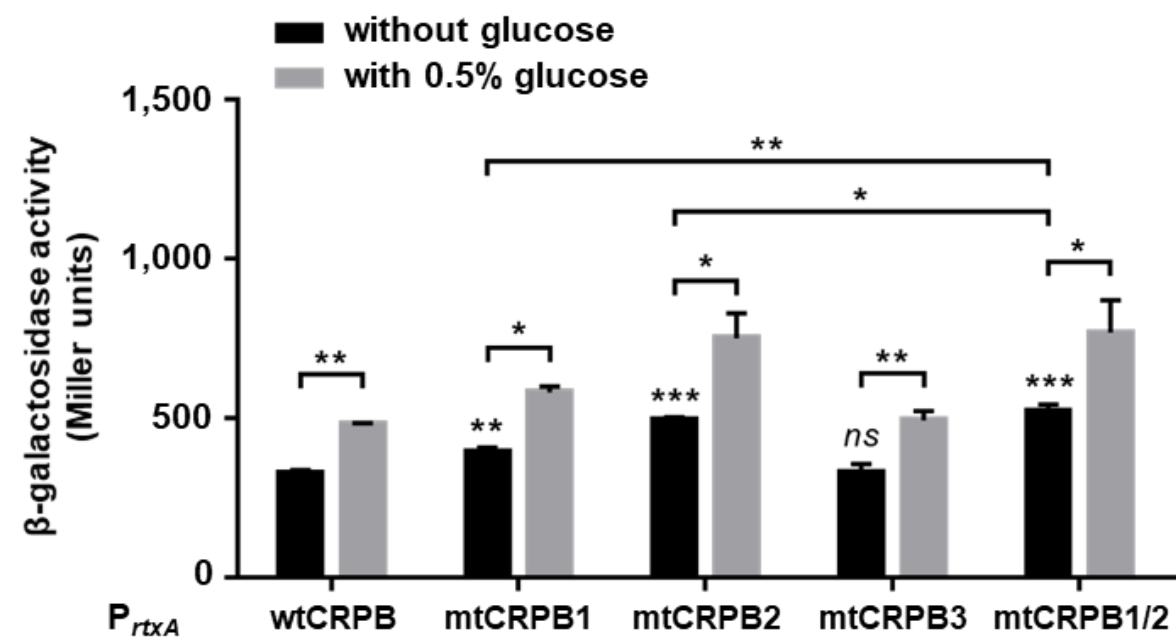

Figure S2. Lee *et al.*

Supplement: FIG S2 [file mBio.00723-20-sf002.pdf]

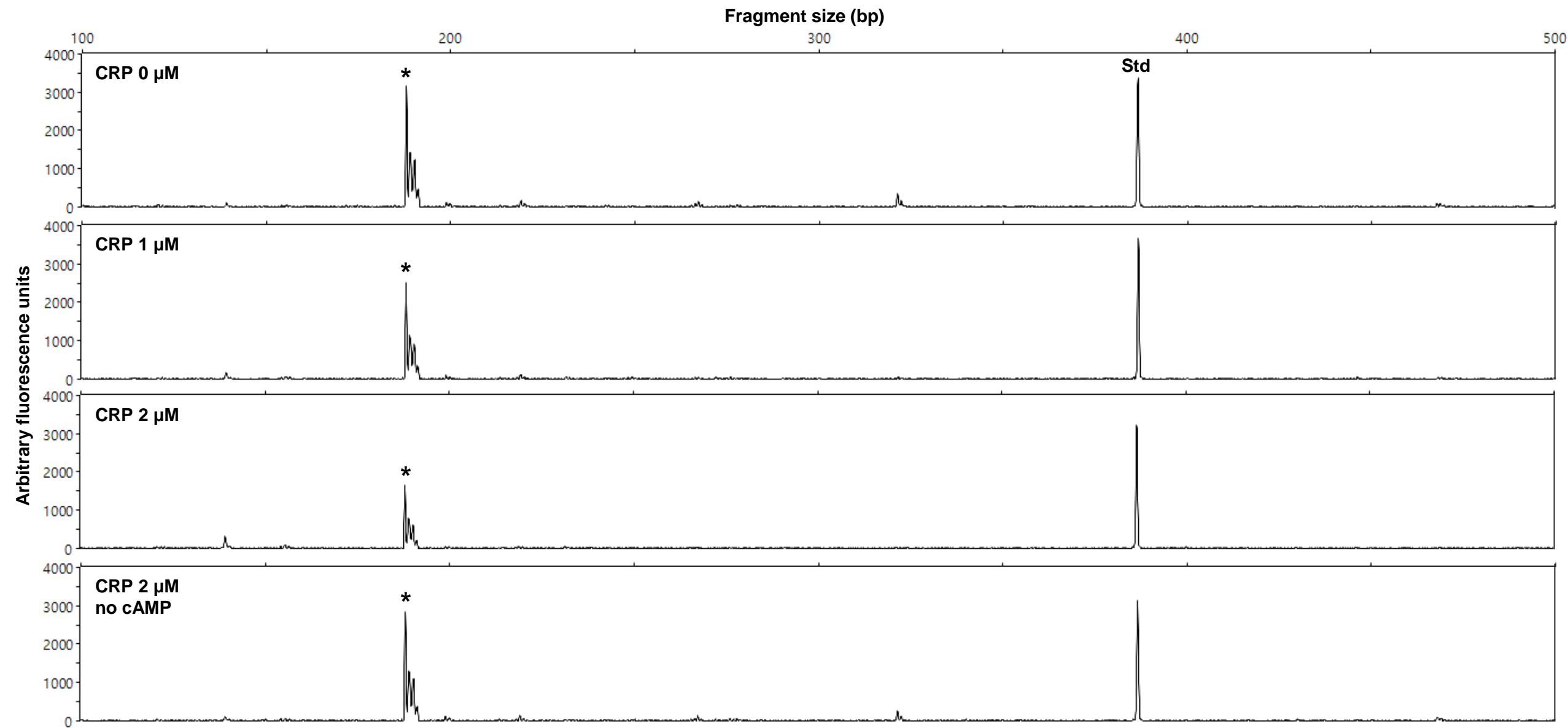

Figure S3. Lee *et al.*

Supplement: FIG S3 [file mBio.00723-20-sf003.pdf]

**A**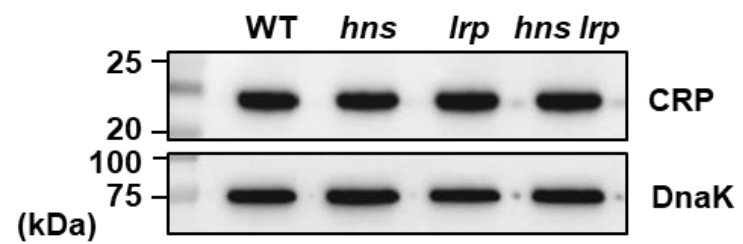**B**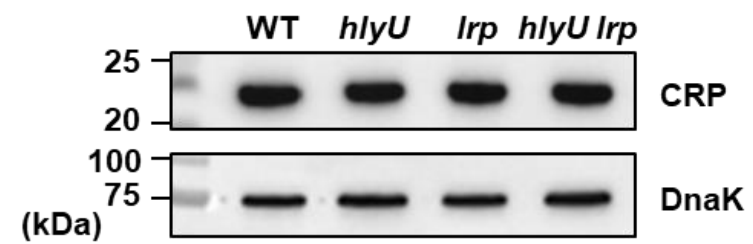

Supplement: FIG S4 [file mBio.00723-20-sf004.pdf]

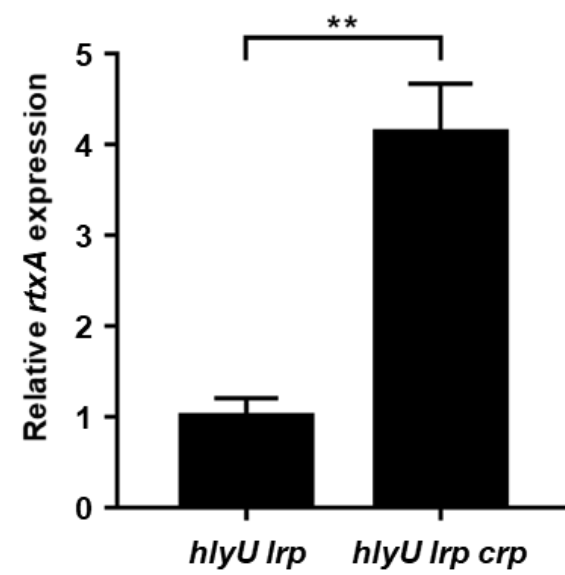

Supplement: FIG S5 [file mBio.00723-20-sf005.pdf]
