# Supplementary material for: A MARTX Toxin rtxA Gene Is Controlled by Host Environmental Signals through a CRP-Coordinated Regulatory Network in Vibrio vulnificus
Source: mBio. 2020 Jul 28;11(4):e00723-20. doi: 10.1128/mBio.00723-20 (PMC7387792; doi:10.1128/mBio.00723-20)
Supplement: TABLE S1 [file mBio.00723-20-st001.docx]

**Table S1. Bacterial strains and plasmids used in this study**

| **Strain or plasmid** | **Relevant characteristics^a^** | **Reference or source** |
| --- | --- | --- |
| **Bacterial strains** |  |  |
| *V. vulnificus* |  |  |
| MO6-24/O | Wild type; clinical isolate; virulent | Laboratory collection |
| ZW181 | MO6-24/O with Δ*lrp* | This study |
| EJ151 | MO6-24/O with Δ*hns* | (1) |
| ZW141 | MO6-24/O with Δ*hlyU* | (2) |
| MO6Δ*lacZ* | MO6-24/O with Δ*lacZ* | (3) |
| DI0201 | MO6-24/O with Δ*crp* | (4) |
| ZW191 | MO6-24/O with Δ*hns* Δ*lrp* | This study |
| ZW192 | MO6-24/O with Δ*hlyU* Δ*lrp* | This study |
| ZW193 | MO6-24/O with Δ*lrp* Δ*lacZ* | This study |
| ZW194 | MO6-24/O with Δ*crp* Δ*lacZ* | This study |
| ZW195 | MO6-24/O with Δ*hlyU* Δ*lrp* Δ*crp* | This study |
| GR192 | MO6-24/O with Δ*toxR* | This study |
| JK093 | MO6-24/O with Δ*iscR* | (5) |
| JK131 | MO6-24/O with Δ*aphA* | (6) |
| JR312 | MO6-24/O with Δ*aphB* | (7) |
| MO6_rpoS | MO6-24/O with Δ*rpoS* | (8) |
| HS03 | MO6-24/O with Δ*smcR*::*nptI*; Km^r^ | (9) |
| ZW201 | MO6-24/O with Δ*lacZ*; ΔP*_rtxA_*::*nptI* | This study |
|  |  |  |
| *E. coli* |  |  |
| S17-1 λ*pir* | Tc::Mu-Km::Tn7;Tp^r^ Sm^r^; host for π-requiring plasmids; conjugal donor | (10) |
| BL21 (DE3) | *F*^-^, *ompT*, *hsdS* (r_B_^-^, m_B_^-^), *gal dcm* (DE3) | Laboratory collection |
|  |  |  |
| **Plasmids** |  |  |
| pDM4 | Suicide vector; R6K γ ori *sacB*; *oriT* of RP4; Cm^r^ | (11) |
| pBS0907 | pDM4 with Δ*crp*; Cm^r^ | (12) |
| pZW1817 | pDM4 with Δ*lrp*; Cm^r^ | This study |
| pGR1907 | pDM4 with Δ*toxR*; Cm^r^ | This study |
| pJH0311 | 0.3-kb MCS of pUC19 cloned into pCOS5; Ap^r^, Cm^r^ | (13) |
| pKK1502 | pJH0311 with *crp*; Ap^r^, Cm^r^ | (2) |
| pZW1818 | pJH0311 with *lrp*; Ap^r^, Cm^r^ | This study |
| pHK0201 | pRSET A with *crp*; Ap^r^ | (4) |
| pZW1610 | pProEX-HTa with *hlyU*; Ap^r^ | (14) |
| pET-28a(+) | His_6_-tag fusion protein expression vector; Km^r^ | Novagen |
| pKK1636 | pET-28a(+) with *hns*; Km^r^ | (1) |
| pZW1903 | pET-28a(+) with *lrp*; Km^r^ | This study |
| pRKΩlacZ | pRK415 derivative containing promoterless *lacZ*; Tc^r^ | (15) |
| pZW1517 | pRKΩlacZ with P*_rtxA_*; Tc^r^ | This study |
| pZW1930 | pRKΩlacZ with P*_rtxA_* carrying mutated CRP-binding sequence 1; Tc^r^ | This study |
| pZW1931 | pRKΩlacZ with P*_rtxA_* carrying mutated CRP-binding sequence 2; Tc^r^ | This study |
| pZW1936 | pRKΩlacZ with P*_rtxA_* carrying mutated CRP-binding sequence 3; Tc^r^ | This study |
| pZW2001 | pRKΩlacZ with P*_rtxA_* carrying mutated CRP-binding sequence 1/2; Tc^r^ | This study |
| pUC4K | pUC4 with the *nptI* gene; Ap^r^, Km^r^ | (16) |
| pMZtc | pDM4 with promoterless *lacZ* for transcriptional fusion | (17) |
| pZW2010 | pMZtc with P*_rtxA_*; Cm^r^ | This study |
| pZW1930 | pMZtc with P*_rtxA_* carrying mutated CRP-binding sequence 1; Cm^r^ | This study |
| pZW1931 | pMZtc with P*_rtxA_* carrying mutated CRP-binding sequence 2; Cm^r^ | This study |
| pZW1936 | pMZtc with P*_rtxA_* carrying mutated CRP-binding sequence 3; Cm^r^ | This study |
| pZW2001 | pMZtc with P*_rtxA_* carrying mutated CRP-binding sequence 1/2; Cm^r^ | This study |

^a^ Sm^r^, streptomycin-resistant; Cm^r^, chloramphenicol-resistant; Ap^r^, ampicillin-resistant; Km^r^, kanamycin-resistant; Tc^r^, tetracycline-resistant.

**References**

1. Choi G, Jang KK, Lim JG, Lee ZW, Im H, Choi SH. 2020. The transcriptional regulator IscR integrates host-derived nitrosative stress and iron starvation in activation of the *vvhBA* operon in *Vibrio vulnificus*. J Biol Chem doi:10.1074/jbc.RA120.012724.

2. Jang KK, Lee ZW, Kim B, Jung YH, Han HJ, Kim MH, Kim BS, Choi SH. 2017. Identification and characterization of *Vibrio vulnificus plpA* encoding a phospholipase A_2_ essential for pathogenesis. Journal of Biological Chemistry 292:17129-17143.

3. Baek CH, Kim KS. 2003. *lacZ*- and *aph*-based reporter vectors for *in vivo* expression technology. Journal of Microbiology and Biotechnology 13:872-880.

4. Choi HK, Park NY, Kim DI, Chung HJ, Ryu S, Choi SH. 2002. Promoter analysis and regulatory characteristics of *vvhBA* encoding cytolytic hemolysin of *Vibrio vulnificus*. Journal of Biological Chemistry 277:47292-47299.

5. Lim JG, Choi SH. 2014. IscR Is a global regulator essential for pathogenesis of *Vibrio vulnificus* and induced by host cells. Infection and Immunity 82:569-578.

6. Lim JG, Park JH, Choi SH. 2014. Low cell density regulator AphA upregulates the expression of *Vibrio vulnificus iscR* gene encoding the Fe-S cluster regulator IscR. Journal of Microbiology 52:413-421.

7. Jeong HG, Choi SH. 2008. Evidence that AphB, essential for the virulence of *Vibrio vulnificus*, is a global regulator. Journal of Bacteriology 190:3768-3773.

8. Kim JA, Lee MA, Jung YC, Jang BR, Lee KH. 2018. Repression of VvpM protease expression by quorum sensing and the cAMP-cAMP receptor protein complex in *Vibrio vulnificus*. Journal of Bacteriology 200.

9. Kim BS, Jang SY, Bang YJ, Hwang J, Koo Y, Jang KK, Lim D, Kim MH, Choi SH. 2018. QStatin, a selective inhibitor of quorum sensing in *Vibrio* species. mBio 9.

10. Simon R, Priefer U, Puhler A. 1983. A broad host range mobilization system for *in vivo* genetic engineering: Transposon mutagenesis in Gram-negative bacteria. Nature Biotechnology 1:784-791.

11. Milton DL, OToole R, Horstedt P, WolfWatz H. 1996. Flagellin A is essential for the virulence of *Vibrio anguillarum*. Journal of Bacteriology 178:1310-1319.

12. Kim BS, Hwang J, Kim MH, Choi SH. 2011. Cooperative regulation of the *Vibrio vulnificus nan* gene cluster by NanR protein, cAMP receptor protein, and *N*-acetylmannosamine 6-phosphate. Journal of Biological Chemistry 286:40889-40899.

13. Goo SY, Lee HJ, Kim WH, Han KL, Park DK, Lee HJ, Kim SM, Kim KS, Lee KH, Park SJ. 2006. Identification of OmpU of *Vibrio vulnificus* as a fibronectin-binding protein and its role in bacterial pathogenesis. Infection and Immunity 74:5586-5594.

14. Lee ZW, Kim BS, Jang KK, Bang YJ, Kim S, Ha NC, Jung YH, Lee HJ, Han HJ, Kim JS, Kim J, Sahu PK, Jeong LS, Kim MH, Cho SH. 2019. Small-molecule inhibitor of HIyU attenuates virulence of *Vibrio* species. Scientific Reports 9.

15. Park DK, Lee KE, Baek CH, Kim IH, Kwon JH, Lee WK, Lee KH, Kim BS, Choi SH, Kim KS. 2006. Cyclo(Phe-Pro) modulates the expression of *ompU* in *Vibrio* spp. Journal of Bacteriology 188:2214-2221.

16. Oka A, Sugisaki H, Takanami M. 1981. Nucleotide sequence of the kanamycin resistance transposon Tn903. J Mol Biol 147:217-26.

17. Kim IH, Kim SY, Park NY, Wen Y, Lee KW, Yoon SY, Jie H, Lee KH, Kim KS. 2018. Cyclo-(L-Phe-L-Pro), a quorum-sensing signal of *Vibrio vulnificus*, induces expression of hydroperoxidase through a ToxR-LeuO-HU-RpoS signaling pathway to confer resistance against oxidative stress. Infection and Immunity 86.
