# Supplementary material for: A MARTX Toxin rtxA Gene Is Controlled by Host Environmental Signals through a CRP-Coordinated Regulatory Network in Vibrio vulnificus
Source: mBio. 2020 Jul 28;11(4):e00723-20. doi: 10.1128/mBio.00723-20 (PMC7387792; doi:10.1128/mBio.00723-20)
Supplement: TABLE S2 [file mBio.00723-20-st002.docx]

**Table S2. Oligonucleotides used in this study**

| **Oligonucleotide** | **Oligonucleotide sequence, 5’** **→3’^a, b^** | **Use** |
| --- | --- | --- |
| **For mutant construction** | | |
| LRPD-F1 | AGCTCAGGTTACCCGCATGCCAGCTTGAGGTTCTTTTACC | Deletion of *lrp* ORF |
| LRPD-R1 | CAACGTAGGATCCCACGCGCTTAGAGAGTTC |  |
| LRPD-F2 | GCGCGTGGGATCCTACGTTGTAATGGAAGAAG |  |
| LRPD-R2 | CTCGAGTACGCGTCACTAGTATATCTCCACCCCATGAGG |  |
| TOXRD-F1 | GAGCTCAGGTTACCCGCATGGAGATGTTGGTCTAAGCG | Deletion of *toxR* ORF |
| TOXRD-R1 | ATCCGGATCCCGTTACGAGTTAACACCTC |  |
| TOXRD-F2 | ACTCGTAACGGGATCCGGATGCCTTCTATTAGGC |  |
| TOXRD-R2 | CGACCCTCGAGTACGCGTCAGTGATGACTGTCACCATATAG |  |
| **For mutant complementation** | | |
| LRPC-F | GAGGATCCCCGGGTACCTTGGTGACCATGTGAGATA | Amplification of *lrp* ORF |
| LRPC-R | CATGATTACGAATTCGAGCTCAGTAACTGAAACATTCCGAG |  |
| **For protein overexpression** | | |
| LRPP-F | GTTTAACTTTAAGAAGGAGATATACCATGGTAGATAACTACAAAAAGCC | Amplification of *lrp* ORF |
| LRPP-R | CAGTGGTGGTGGTGGTGGTGACGTGTTTTAATCACAAGTTG |  |
| **For EMSA and DNase I protection assay** | | |
| PrtxA_P1-F | GTTAAGTTCGTGATAAGAGACCAC | Amplification of P*_rtxA_* regulatory region, Probe 1 |
| PrtxA_P1-R | CACACAATGAAGACCAATAAACG |  |
| PrtxA_P2-F | CGTTTATTGGTCTTCATTGTGTG | Amplification of P*_rtxA_* regulatory region, Probe 2 |
| PrtxA_P2-R | TTTCAGCCATTACGCCATT |  |
| Plrp-F | AATGAGCTCTGAAAAACCGATGCCT | Amplification of P*_lrp_* regulatory region |
| Plrp-R | TTTACTAGTTGGAGAAAGCCCCACG |  |
| PhlyU-F | CAAGAGCTCGACTCGACACAAAGT | Amplification of P*_hlyU_* regulatory region |
| PhlyU-R | AGACTAGTTCATGTGTTGGTCCTCTAG |  |
| **For reporter construction** | | |
| PrtxAZ-F | CTGCAGGAATCAAATAAAATGGCGG | Amplification of P*_rtxA_* regulatory region |
| PrtxAZ-R | GGATCCATTTTTTTGATCCTGGCCTAC |  |
| PrtxA_UP-F | GAGCTCAGGTTACCCGCATGCGTAGACCACGAAGGACC | Amplification of P*_rtxA_* upstream region |
| PrtxA_UP-R | ACAAGGCGCACCATTTATTCTA |  |
| PrtxA_DW-F | ATGGCGAATGGCGTAATGG | Amplification P*_rtxA_* downstream region |
| PrtxA_DW-R | CGACCCTCGAGTACGCGTCATTTAGCTCAAAAGCAGGCAG |  |
| nptI_PrtxA-F | GAATAAATGGTGCGCCTTGTGGAAAGCCACGTTGTGTC | Amplification of *nptI* cassette |
| nptI_PrtxA-R | GCCATTACGCCATTCGCCATTTAGAAAAACTCATCGAGCATC |  |
| PrtxAM1-F | CCCTTCTAGATAGATCTTGCATGTAGACCACGAAGGACC | Amplification of P*_rtxA_* upstream region |
| PrtxAM1-R | CAAAGAAGAACTCGAAAAAATACAAAAAGCAAAC |  |
| PrtxAM2-F | GTTTGCTTTTTGTATTTTTTCGAGTTCTTCTTTG | Amplification of P*_rtxA_* |
| PrtxAM2-R | CTCAGGTTACCCGCATGATTTTTTTGATCCTGGCCTAC |  |
| **For site-directed mutagenesis** | | |
| CRPB1_mt-F | AATACAAAACCGCGTCAAGCGTTCATTGCCGTCCATAATGAAATTA | Site-directed mutagenesis of CRP-binding sequence 1 |
| CRPB1_mt-R | TAATTTCATTATGGACGGCAATGAACGCTTGACGCGGTTTTGTATT |  |
| CRPB2_mt-F | CAAATGAATGATGCAGCATTCGTTAAGATGTAATCAAGGT | Site-directed mutagenesis of CRP-binding sequence 2 |
| CRPB2_mt-R | ACCTTGATTACATCTTAACGAATGCTGCATCATTCATTTG |  |
| CRPB3_mt-F | TCAAGGGCCTACGTCCATGAAGATGGAATTGAG | Site-directed mutagenesis of CRP-binding sequence 3 |
| CRPB3_mt-R | CTCAATTCCATCTTCATGGACGTAGGCCCTTGA |  |
| **For *in vitro* transcription** | | |
| PrtxA_ivt-R | CACCTTTATTGTAGTTAACACTAGATACTG | *in vitro* transcription of *rtxA* |
| **For qRT-PCR analysis** | | |
| RTXA_qRT-F | TAGCGGCGACAATGAAACCT | qRT-PCR |
| RTXA_qRT-R | CCCATCACCGCAAGGGTATT |  |
| LRP_qRT-F | GGGGCTTTCTCCAACTCCAT |  |
| LRP_qRT-R | GCAACGAGGCGTCTAGGTAT |  |
| HLYU_qRT-F | CATGGCCAATGAAAGACGCC |  |
| HLYU_qRT-R | ACCATGCCAGATGCTGAGAC |  |

^a^ The oligonucleotides were designed using the *V. vulnificus* MO6-24/O genomic sequence (GenBank^TM^ accession number CP002469 and CP002470, www.ncbi.nlm.nih.gov).

^b^ Regions of oligonucleotides not complementary to the corresponding genes are underlined.
