# Supplementary material for: A MARTX Toxin rtxA Gene Is Controlled by Host Environmental Signals through a CRP-Coordinated Regulatory Network in Vibrio vulnificus
Source: mBio. 2020 Jul 28;11(4):e00723-20. doi: 10.1128/mBio.00723-20 (PMC7387792; doi:10.1128/mBio.00723-20)
Supplement: TEXT S1 [file mBio.00723-20-s0001.docx]

**Text S1. Supplemental Methods**

**Construction of chromosomal *rtxA*-*lacZ* transcriptional fusion reporter strains.** To construct the chromosomal *rtxA*-*lacZ* transcriptional fusion reporter strain, the *lacZ* mutant with ΔP*_rtxA_*::*nptI*, ZW201, was first generated by deletion of the P*_rtxA_* region and insertion of the *nptI* cassette conferring kanamycin resistance. Pairs of primers, PrtxA_UP-F and -R, or PrtxA_DW-F and -R were used for amplification of the upstream region of P*_rtxA_* and the downstream region of P*_rtxA_*, respectively (Table S2). A pair of primers, nptI_PrtxA-F and -R was used for amplification of the *nptI* cassette in conjunction with pUC4K (1). The resulting DNA fragments were cloned into SpeI-SphI-digested pDM4 (2) using the NEBuilder^®^ HiFi DNA Assembly Master Mix (New England BioLabs, NEB) to create pZW2005. *E. coli* S17-1 λ*pir* strain (3) containing pZW2005 was used as a conjugal donor to the *lacZ* mutant to generate the *lacZ* mutant with ΔP*_rtxA_*::*nptI*, ZW201 (Table S1).

Next, to construct the *rtxA*-*lacZ* transcriptional fusion, pairs of primers, PrtxAM1-F and PrtxAM1-R or PrtxAM2-F and PrtxAM2-R were used for amplification of the upstream region of P*_rtxA_* and P*_rtxA_* region (-526 to +227 relative to the transcription start site of *rtxHCA*), respectively (Table S2). The resulting DNA fragments were cloned into SphI-digested pMZtc (4), which carries promoterless *lacZ* (195-bp upstream from the translation start site of *lacZ*) for a single crossover, to create pZW2010 (Table S1). Similarly, pZW2011, pZW2012, pZW2013, and pZW2014 with the P*_rtxA_* region carrying either mtCRPB1, mtCRPB2, mtCRPB3, or mtCRPB1/2, were constructed (Table S1). *E. coli* S17-1 λ*pir* strain (3) containing pZW2010, pZW2011, pZW2012, pZW2013, or pZW2014 was used as a conjugal donor to ZW201. A single crossover was obtained by selecting colonies resistant to kanamycin and chloramphenicol.

**References**

1. Oka A, Sugisaki H, Takanami M. 1981. Nucleotide sequence of the kanamycin resistance transposon Tn903. J Mol Biol 147:217-26.

2. Milton DL, OToole R, Horstedt P, WolfWatz H. 1996. Flagellin A is essential for the virulence of *Vibrio anguillarum*. Journal of Bacteriology 178:1310-1319.

3. Simon R, Priefer U, Puhler A. 1983. A broad host range mobilization system for *in vivo* genetic engineering: Transposon mutagenesis in Gram-negative bacteria. Nature Biotechnology 1:784-791.

4. Kim IH, Kim SY, Park NY, Wen Y, Lee KW, Yoon SY, Jie H, Lee KH, Kim KS. 2018. Cyclo-(L-Phe-L-Pro), a quorum-sensing signal of *Vibrio vulnificus*, induces expression of hydroperoxidase through a ToxR-LeuO-HU-RpoS signaling pathway to confer resistance against oxidative stress. Infection and Immunity 86.
